# Advances in osteogenic niche engineering: integrating topography and electrical cues for bone repair and regeneration

**DOI:** 10.1186/s12951-026-04852-x

**Published:** 2026-08-01

**Authors:** Jingyan Huang, Dongheng Lu, Yixuan Liu, Taras Vladimirovich Furtsev, Peng Yu, Chenye Hou, Xiaoshuang Wang, Weichang Li, Yan Wang

**Affiliations:** 1 Hospital of Stomatology, Guanghua School of Stomatology, Guangdong Provincial Key Laboratory of Stomatology, Sun Yat-sen University, Guangzhou, 510055 China; 2 Department of Orthopedic Dentistry, Faculty of Dentistry, Krasnoyarsk State Medical University, Krasnoyarsk, 660022 Russia; 3 School of Materials Science and Engineering, National Engineering Research Center for Tissue Restoration and Reconstruction, South China University of Technology, Guangzhou, 510641 China

**Keywords:** Topography, Electrical Stimulation, Bone Regeneration

## Abstract

Bone defects caused by aging and trauma present significant clinical challenges, driving intense research into bone regeneration strategies. Cellular behaviour during bone repair is critically regulated by the sustainable biophysical cues of the bone niche, particularly topography and electrical signals. Recent advances in biomaterial engineering now enable the precise modulation of these microenvironmental factors—through tuneable topographical and electroactive interfaces—to promote osteogenesis. This review examines emerging evidence on the individual and synergistic roles of surface topography and electrical stimulation in bone regeneration, highlighting their potential as non-pharmacological therapeutic approaches.

**Graphical Abstract:**

**Figure:**
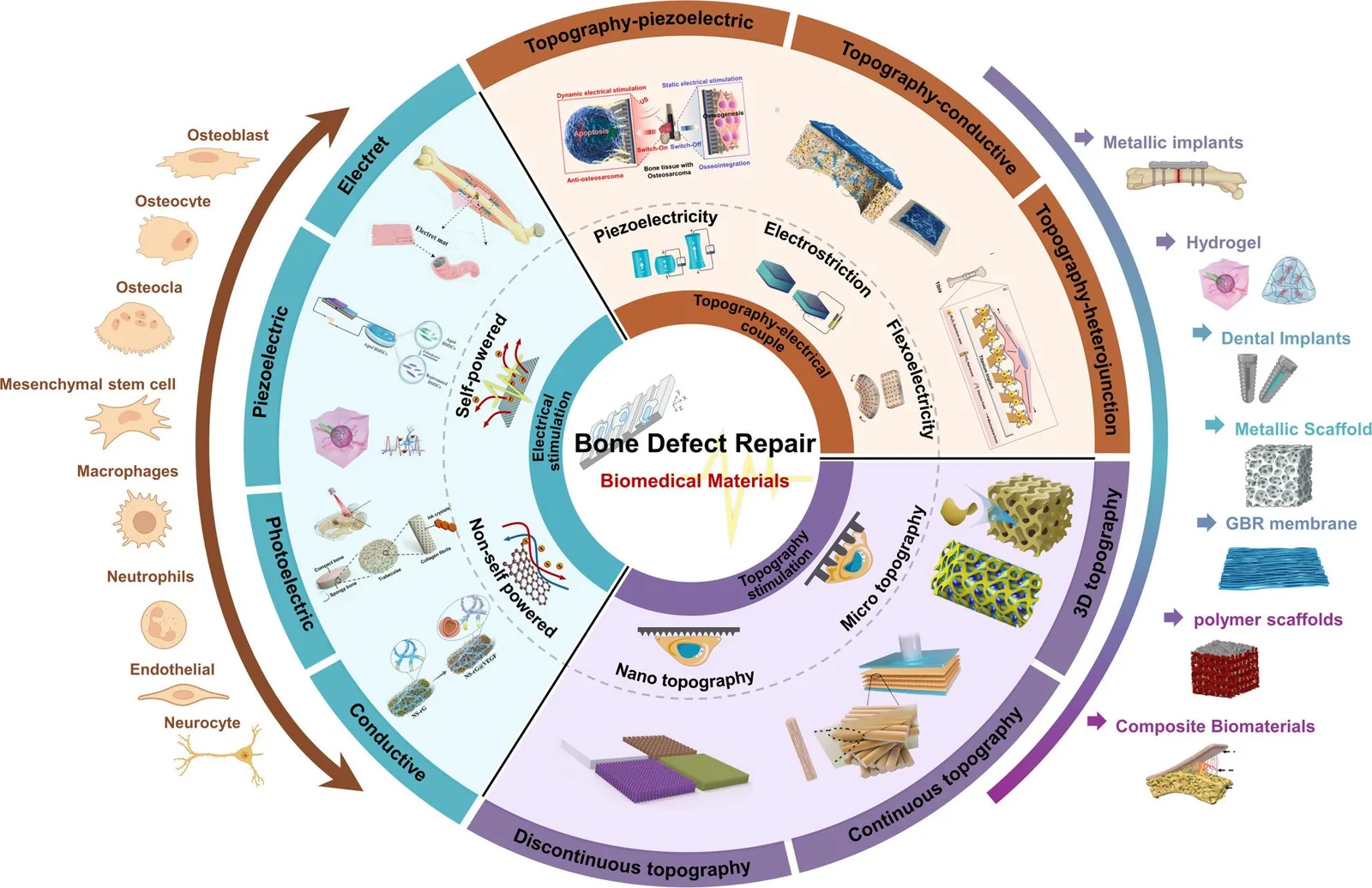
Schematic overview of osteogenic niche engineering based on synergistic topographical and electrical cues to orchestrate bone regeneration

## Introduction

As the global population ages, the proportion of individuals aged 65 and over is predicted to rise to approximately 20% of the global population by the year 2050 [1]. The aging population, coupled with changes in osteogenic mechanical behaviour with aging and disease, has led to a marked increase in bone injuries and defects, having profound consequences for individuals’ quality of life [2]. Bone defects are focal losses of bone tissue that exceed the bone’s intrinsic capacity for spontaneous repair. Various aetiologies, including trauma, osteoporosis, debridement in bone infection, and resection in bone tumours, can lead to persistent nonunion without surgical intervention [3, 4], which exerted a considerable financial burden on public healthcare systems worldwide [5, 6]. Consequently, addressing bone defects has become a significant and pressing challenge in clinical practice. Specifically, autologous bone grafting remains the “gold standard” for bone defects but is limited by donor‑site morbidity and finite harvest volume [7]. Allogeneic grafts circumvent autograft shortages but carry risks of immune rejection and disease transmission [8]. To overcome the limitations of current treatment strategies, a diverse array of bone repair materials has been developed to meet pressing clinical needs. These materials encompass biodegradable scaffolds, metallic implants, guided bone regeneration membranes and composite biomaterials. Augmenting the osteogenic capacity and regenerative efficacy of bone-grafting materials represents a pivotal therapeutic strategy for osseous defects.

As research continues to unravel the bone niche’s role in regeneration, bioengineering—an ever-evolving discipline—persistently advances novel approaches to replicate its critical features. The bone niche, also known as the bone microenvironment, is a complex network of cell types and acellular factors that supports normal homeostasis [9, 10].

The cellular components of the bone niche mainly include osteoblasts (OBs), mesenchymal stromal cells (MSCs), endothelial cells (ECs), fibroblasts, pericytes, nerve cells and smooth muscle cells. Among these cells, osteoblasts, osteocytes, and osteoclasts are the three major cell types comprising the bone niche, whose dynamic equilibrium profoundly governs bone remodelling via regulating the balance of bone resorption and bone regeneration [11]. In addition, the bone vasculature sustains the bone niche by supplying oxygen, nutrients and progenitors and also provides angiocrine signals, mainly from endothelial cells. Herein, a specialized subset—type H endothelial cells—closely interacts with osteogenic precursors and secretes factors such as VEGF, HIF-1α, and Notch ligands to coordinate vessel assembly and stimulate bone formation [12, 13]. As an innervation-rich tissue, the skeletal nervous system plays a critical role in regulating bone homeostasis [14]. Nerve fibres and Schwann cells secrete neurotrophins and neuropeptides to enhance angiogenesis, recruit osteogenic progenitors, and regulate osteoblast/osteoclast dynamics [15, 16]. Moreover, immune cells first drive acute inflammation after bone injury, with neutrophils and M1 macrophages releasing cytokines to recruit MSCs and osteoprogenitors. As repair progresses, M1 macrophages convert to M2 macrophages that secrete IL-4, IL-10, and TGF-β to boost angiogenesis and MSC differentiation, enabling bone remodelling [17, 18]. Anchored by the cell niche, the coordinated interplay of multiple cellular microenvironments collectively regulates the biological behaviours and fate decisions underlying bone defect repair [19, 20]. Therefore, orchestrating the coordinated coupling of the osteogenic niche microenvironment—including immune cells, neural cells, endothelial cells and osteoblasts/osteoclasts—to promote more efficient and effective osteogenesis is a central objective in the development of bioengineered bone repair materials.

Apart from the cellular contributions, the bone niche includes biochemical factors (soluble factors and oxygen tension), the ECM (extracellular matrix) and biophysical regulators. Clarifying the underlying mechanism of action of each regulator would help direct the design and fabrication of improved scaffolds for bone regeneration. While the role of chemical cues in bone regeneration is well appreciated, the cues presented by the physical microenvironment are increasingly recognised as important regulators of osteogenic differentiation, contributing to stable and controllable characteristics. ECM, a highly dynamic non-cellular three-dimensional network, serves as an anchoring structure by mechanically linking the extracellular and intracellular worlds and provides structural support, biochemical signalling, and mechanical cues to resident cells [21–23]. The biophysical cues, including matrix elasticity, hydrostatic pressure, shear stress, topographical features and electrical conductivity, synergistically dictate cell fate by engaging mechanotransduction pathways and bioelectric signalling [24–28].

In recent years, a variety of bone repair materials that biomimetically replicate bone tissue-specific topographical features through the engineering of substrates and 3D scaffolds have been developed [29]. Nanoscale architectures, such as ZrO₂/TiO₂ nanotube arrays, maximize integrin activation (evidenced by paxillin recruitment) to enhance MSC adhesion and proliferation [30, 31]. Surface micro/nanotopography (MNT) directly enhances osteogenic differentiation by inducing endoplasmic reticulum (ER) stress and upregulating GRP78/BiP, with nanotube dimensions (30–100 nm) modulating the severity of ER morphological distortion and osteogenic efficiency. These preliminary findings underscore topography-driven mechanotransduction as a pivotal factor in bone-regenerative biomaterial design [32]. These bone repair materials with well-designed nano/microtopography not only possess excellent mechanical properties but also substantially accelerate the bone healing process [33]. Also, numerous experimental studies have demonstrated that bone repair materials mimicking the electrophysiological properties of the bone niche, along with the application of electrical stimulation, constitute a viable strategy for enhancing bone healing rates [3].

Previous reviews have extensively discussed either surface topography or electrical stimulation as individual strategies for enhancing bone regeneration. Moreover, the application of electroactive materials and electrical stimulation in promoting osteogenesis has been summarized [34–36]. However, these two research areas have largely been treated independently. Additionally, existing reviews summarizing the synergetic biological effects of topography and electrical cues focus more on the neural differentiation of stem cells and nerve regeneration [37, 38], while the synergistic effect and mechanisms of these two cues on bone repair are rarely systematically elaborated. In contrast, this review provides a unified perspective on the combined roles of topographical and electrical cues as interconnected biointerfacial regulators. We propose a conceptual framework in which topography-induced mechanotransduction and electrically mediated signalling converge on physical properties and shared pathways, such as ion-channel activation and downstream gene regulation. Furthermore, this review adopts a parameter-oriented approach to compare key interfacial design factors across early-stage studies, facilitating cross-study interpretation. Importantly, we critically evaluate the current evidence supporting synergistic effects and identify key limitations and knowledge gaps, thereby offering forward-looking insights into the rational design of multifunctional and adaptive biointerfaces for bone regeneration.

**Fig. 1:**
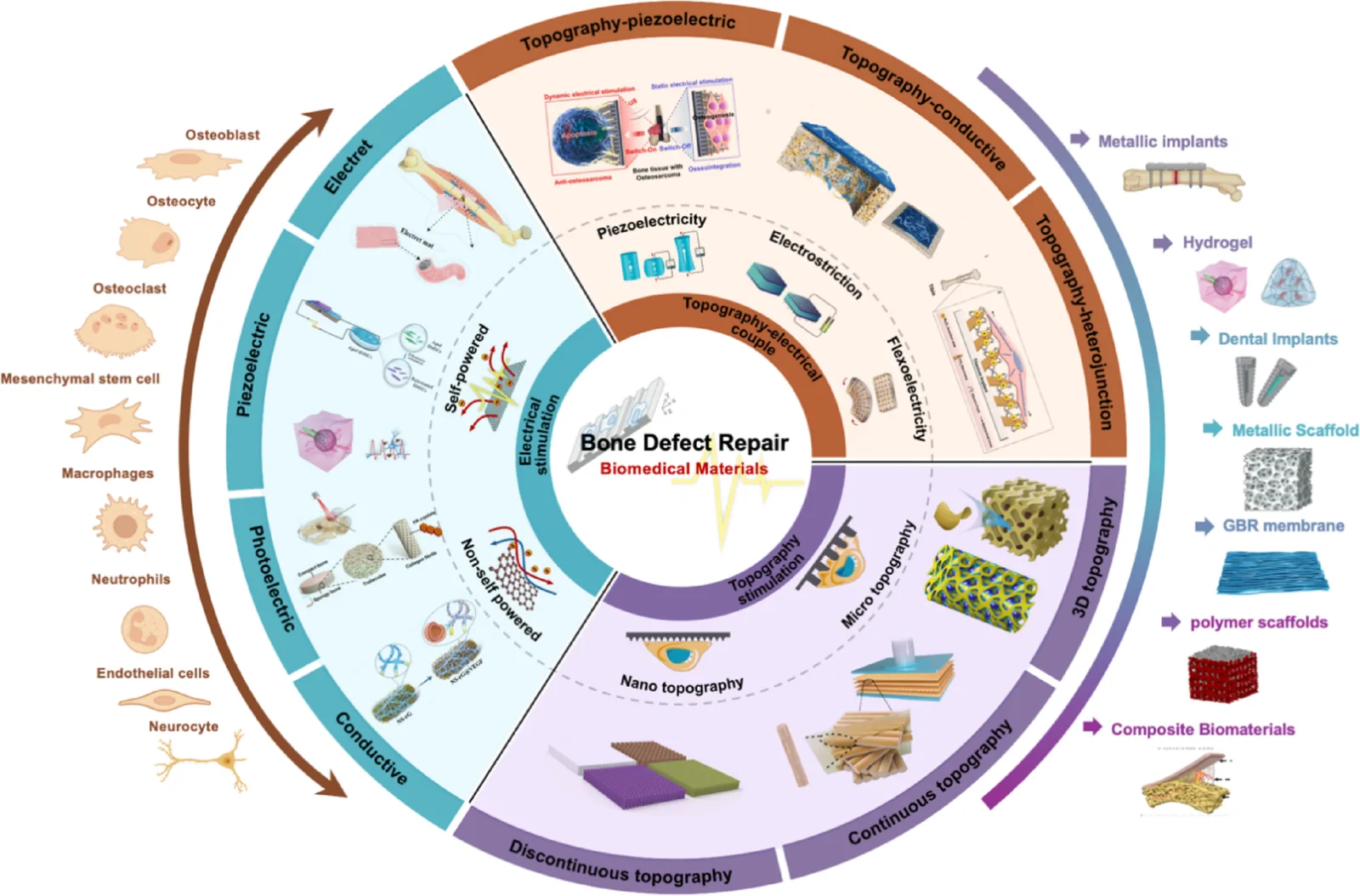
Bone niche engineering through topography and electrical stimulation for bone regeneration. The left panel outlines the diverse possible target cells modulated by signals in bone repair process. The central part illustrates three core material design strategies: (1) Electrical stimulation, encompassing self-powered and non-self powered system to deliver intrinsic or extrinsic electrical cues; (2) Topography stimulation, utilizing nano and micro structures, along with continuous or discontinuous surface patterns, to physically guide cell behavior; (3) Topography and electrical co-stimulation, integrating morphological and electrical features (such as topography-piezoelectric, topography-conductive, and topography-heterojunction designs) may provide synergistic microenvironmental regulation. The right panel summarizes the translation of these biophysical concepts into versatile clinical biomaterials, ranging from hydrogels and membranes to diverse tissue-engineered scaffolds. The integrated strategy represents an emerging direction in biointerfacial engineering, with current evidence largely originating from preclinical studies

In brief, engineering bone repair materials with bionic topographical and electrophysiological properties in the osteogenic niche enabling coordinated modulation of multiple cellular components constitutes a critical research frontier in contemporary bone tissue engineering. In this review, the main focus is to summarize recent developments on bone repairing materials with topography and electrical stimulation cues, elucidating the single and synergistic effects and the intrinsic mechanisms. (Fig. 1).

## Review methodology and search strategy

The literature search was conducted in PubMed, Scopus, and Web of Science to identify relevant studies published between 2015 and 2025 using keywords such as “electrical signals”, “electrical stimulation”, “topography”, “morphology”, “surface morphology”, “surface topography”, “osteogenesis”, and “osseointegration”. These terms were combined using Boolean operators (AND/OR) to refine the results and target early-stage studies integrating topography and electrical cues for bone repair and regeneration. The inclusion criteria comprised: (1) peer-reviewed original research papers; (2) studies investigating biomaterials for bone defect repair; and (3) research involving relevant in vitro or in vivo biological evaluations. The exclusion criteria comprised: (1) conference abstracts, book chapters, unpublished papers or preprints; (2) studies focusing on non-biomedical engineering materials or purely theoretical/computational models without biological validation; and (3) articles with insufficient methodological clarity or lacking appropriate control groups.

## Biological basis of bone regeneration

### Osseous cellular ensemble in bone regeneration

Bone regeneration is driven by coordinated activity among MSCs, osteoblast-lineage cells, osteocytes, and osteoclasts [39]. MSCs replenish osteoprogenitors and provide paracrine support [40]; osteoblasts deposit and mineralize the matrix [41, 42]; osteocytes sense matrix strain and fluid-flow changes [43–45]; and osteoclasts resorb aged bone to permit coupled remodelling [46–48]. BMP/WNT signalling and the RANKL/OPG axis together define the minimal biological framework needed to interpret how material cues later influence adhesion, migration, mechanosensing, and differentiation [47, 49–51].

### Dynamics of the cellular osteogenic niche in bone regeneration

**Fig. 2:**
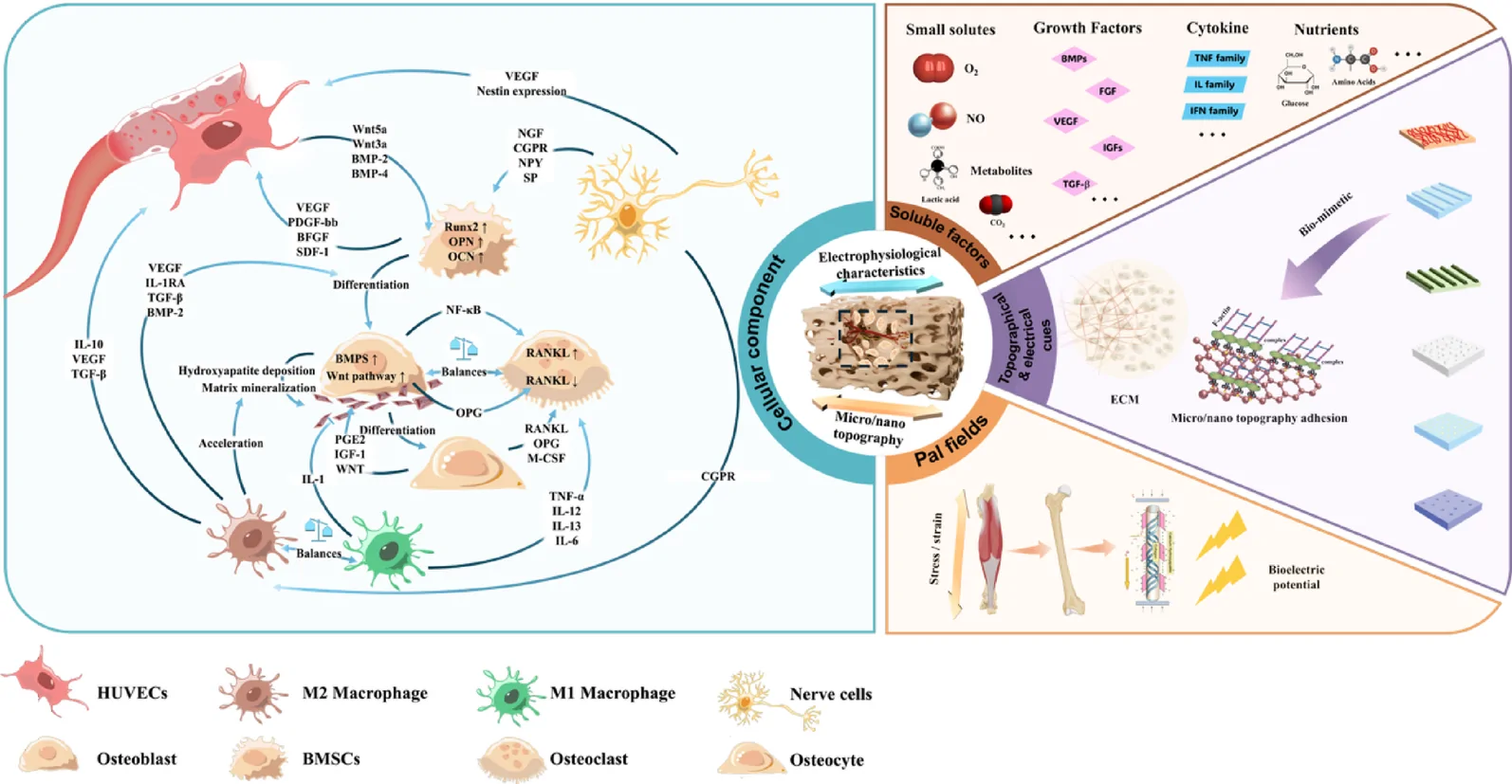
Schematic illustrating the main elements of the osteogenic niche. The niche microenvironment comprises four principal components—adjacent cells, soluble factors, the extracellular matrix, and biophysical signals (such as electrical and mechanical). The osteogenic niche components collectively influence cell fate

A working understanding of the bone niche, including its structural organization, cellular interactions, and biomaterial-responsive microenvironment, has informed recent advances in biomaterial design for bone regeneration [52]. Niche cells coordinate local signals that regulate stem and progenitor cell behaviour according to the regenerative demands of the tissue [53]. Consequently, the sophisticated and harmonious coordination of innervation, immunomodulation, and vascularization within the bone niche has been highlighted during the process of bone regeneration. Beyond cellular interactions, bone regeneration is further influenced by non-cellular niche components, including soluble factors, extracellular matrix architecture, and biophysical stimuli (Fig. 2).

#### Immunomodulation-driven bone regeneration

After injury, inflammatory cells recruit MSCs and osteoprogenitors, whereas a timely transition towards pro-healing macrophage states supports angiogenesis, matrix remodelling, and osteogenic differentiation [54, 55]. Interface-mediated regulation of the immune microenvironment can therefore materially influence osseointegration.

#### Vascularization-driven bone regeneration

Vascular ingrowth supplies oxygen, metabolites, immune cells, and progenitors, while endothelial cells provide angiocrine signals [56, 57]. Type H vessels are especially relevant because they associate with osteoprogenitors and link HIF-1α, VEGF, and Notch signalling to coupled angiogenesis and osteogenesis [12, 13, 58].

#### Innervation-driven bone regeneration

Bone repair is also influenced by neural inputs [59]. Sensory fibres and Schwann-cell-associated signals, including NGF/CGRP-related neurovascular crosstalk [60, 61], regulate MSC, osteoblast, and osteoclast behaviour and coordinate reinnervation with vascular remodelling [62].

### Multidimensional regulatory mechanisms of ECM in bone repair

Acellular Bone Niche Crosstalk in Bone Regeneration Within the acellular compartment, soluble factors and extracellular matrix components shape the biochemical and structural context in which cells perceive and respond to material cues [63]. Collagens, laminins, proteoglycans, and integrin-mediated signalling organize adhesion, growth-factor presentation, and osteogenic signal transduction [64, 65]. Native bone ECM is also hierarchically structured, with collagen–mineral organization and multiscale surface features that influence focal adhesion assembly and lineage specification [66–68].

ECM-mediated regulation operates through multiple complementary mechanisms. Biochemical signalling via specific matrix–receptor interactions constitutes one such pathway. For example, α2β1 integrin binding to type I collagen has been shown to enhance ERK1/2 phosphorylation, a process associated with osteoblast differentiation and mineral deposition [69, 70]. In parallel, laminin-332 interactions with α3β1 integrin activate similar ERK-dependent pathways to promote MSC osteogenesis [71]. These representative examples coexist with broader regulatory networks involving proteoglycans, such as decorin and syndecan-1, which modulate growth factor bioavailability within the niche [65].

The ECM functions as far more than a simple structural scaffold; its intrinsic biochemical composition actively coordinates and regulates cellular responses by binding and presenting growth factors [72]. Beyond existing as freely dissolved molecules within the niche environment as discussed above, soluble growth factors (such as BMP, VEGF, FGF, TGF-β, and PDGF) are stored, localized, and presented through specific binding with ECM macromolecules (including proteoglycans, fibronectin, and other matrix-associated proteins) [72, 73]. By finely regulating the bioavailability and spatiotemporal distribution of these cytokines, these ECM macromolecules play an important regulatory role in tissue development, injury repair, and cellular microenvironment homeostasis [72, 73].

Except for biochemical composition, the precise role of various physical properties in directing cell fate remains a subject of ongoing research due to the continuous improvement of experimental methods and technologies. The relative contribution of micro–nanoscale topography versus bulk mechanics, such as stiffness, in directing cell fate is still not fully resolved, particularly in the context of complex native bone structures composed of mineral and collagen phases. Type I collagen fibrils, with diameters of 50–200 nm, display a 67 nm D-periodic banding pattern and are extensively inter- and intramolecularly cross-linked, providing tensile strength and a template for bone regeneration. Hydroxyapatite nanocrystals are aligned in the 40 nm gap zones of collagen fibrils, occupying a preformed, osmotically crowded, covalently crosslinked organic niche [23, 74]. These physical properties of the ECM, especially the micro/nanostructures, are crucial for cell proliferation and differentiation by affecting cell elongation, deformation and gene expression via mechanically-sensitive proteins [75]. While surface topography offers advantages by directly priming integrin clustering and downstream mechanotransduction cascades, it is essential to recognize that bulk mechanical properties, such as ECM stiffness and elastic modulus, are also critical in directing osteogenesis. Current evidence reveals a highly complex interplay between these physical cues [76]. This hierarchical regulation aligns with observations that ECM-derived mechanical strain synergizes with collagen signalling to amplify osteogenic responses [66, 67, 77]. Collectively, the bone niche leverages the multifaceted properties of the ECM to spatially and temporally coordinate osteoinductive signals, positioning surface topography as a scaffold design priority for bone regeneration.

On the other hand, the electrical properties of the ECM also play a crucial role in the process of bone defect repair. The ECM displays intrinsic piezoelectricity arising from its noncentrosymmetric type I collagen fibrils, in which mechanical deformation of collagen generates surface charges that enhance cell adhesion and proliferation [78, 79]. The piezoelectric effect of bone converts mechanical energy into electrical energy, generating an electrical stimulus that modulates osteoblast attraction and calcium deposition [80, 81]. Numerous experimental studies have demonstrated that bone repair materials mimicking the electrophysiological properties of the ECM, along with the application of electrical stimulation, constitute a viable strategy for enhancing bone healing rates [3]. Therefore, investigating the synergistic interactions between electrical activation and various biomaterials represents a critical research focus in the field of bone tissue engineering [3].

## Topography affects bone regeneration

Topographical patterns can be divided into microstructures and nanostructures. In the context of biomaterial engineering, microstructures are defined as structures with one or more dimensions ≥ 1 µm [33, 82]. Nanostructures, on the other hand, refer to structures with at least one external or internal dimension between 1 and 100 nm [83]. For structures in the 100 nm to 1 μm range, definitions vary among researchers, reflecting the lack of a single, universally accepted boundary between nano- and microstructure materials. Some investigators classify these features as submicronscale, whereas others include them within a broader nanoscale domain, extending the concept of “nanostructures” up to 1,000 nm in at least one dimension [33, 84]. The European Commission defines a “nanomaterial” as a material in which “one or more external dimensions are in the size range 1–100 nm”, whereas the International Organization for Standardization (ISO), in ISO/TS ISO 80004-1:2023, defines “nanoscale” as a size range of “approximately” 1–100 nm and includes nano-objects with sizes larger than 100 nm [83, 85]. Given that nanostructures slightly above 100 nm exhibit similar biological behaviour in bone tissue, the broader definition of nanostructures was adopted for the sake of simplicity and clarity [86].

**Fig. 3:**
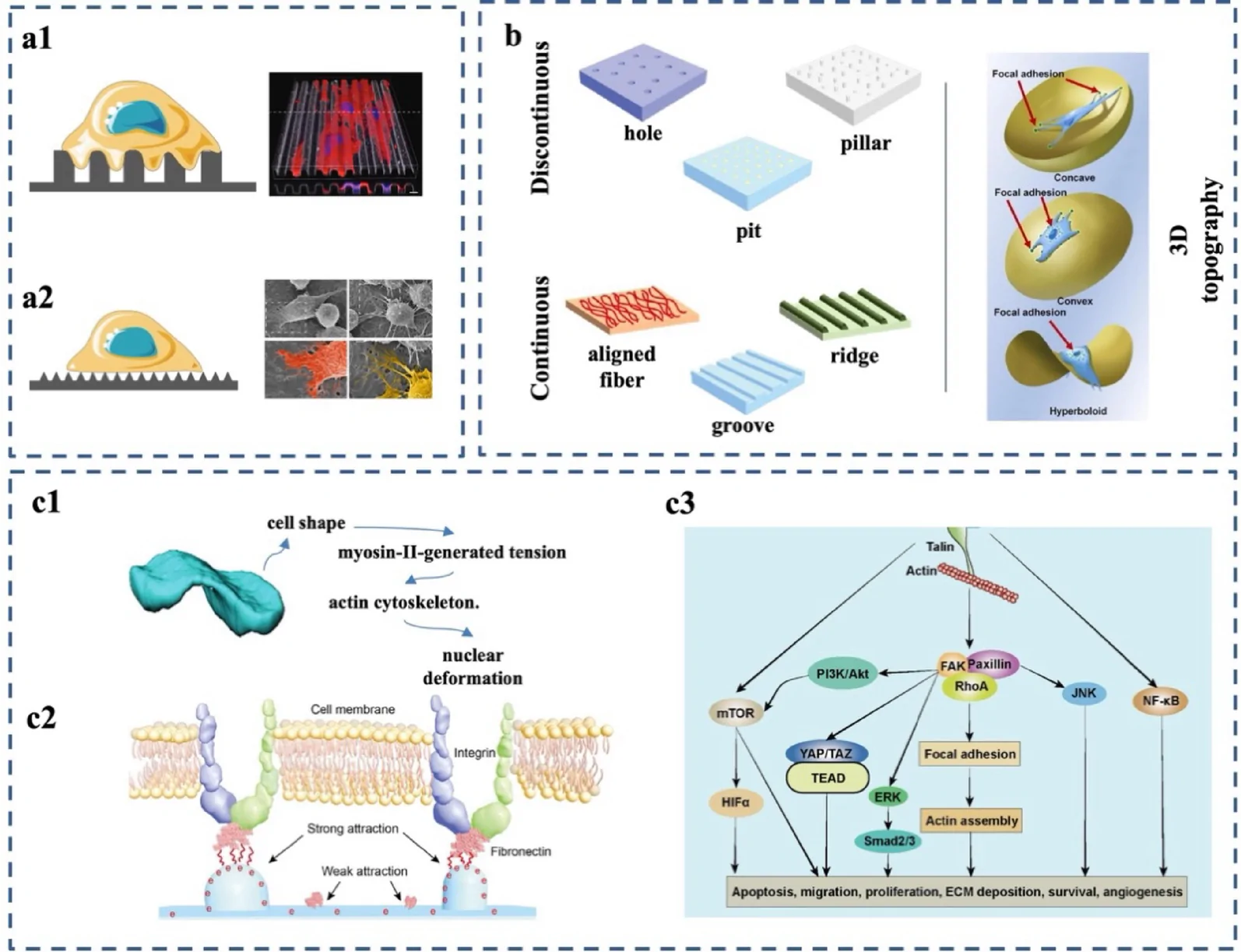
Schematic overview in which micro/nanoscale topographies direct cell behaviour through modulation of morphology and activation of downstream signalling pathways.(**a1**) Schematic illustration of cell deformation induced by microscale topography and 3D reconstruction of cell membranes (red) and nuclei (blue) on microgrooves. Large protrusions of the cell membrane along the groove walls down to the bottom of the grooves were observed [149].(**a2**) Schematic illustration of cell deformation induced by nanoscale topography and SEM of the cellular morphology on different samples. The cells exhibited an irregular shape with cytoplasm spread widely, revealing abundant filopodia, many of which penetrated the pores of the nano/microstructures [153].(**b**) Schematic illustration of the classification of topography types [91, 102, 158].(**c1**) Schematic demonstrating the influence of microtopography on cell behaviour [87].(**c2**) Schematic demonstrating the influence of nanotopography on cell behaviour, showing that transmembrane receptors, such as integrins, can interact with fibronectin adsorbed on the nanotopography, subsequently adopting active or inactive states [158].(**c3**) Schematic illustration of the mechanisms of cellular response and downstream cascades responding to mechanical stimulation [159]

Microstructural topographical patterns have the ability to enhance MSC osteogenesis by affecting tension on the actin cytoskeleton [87]. The regulation of tension within the cytoskeleton is the driving factor for mechanosensitive signalling and gene activity to invoke changes in cell proliferation, migration and differentiation by modulating the nuclear morphology and chromatin architecture [87–89] (Fig. 3a1). On the other hand, nanostructural topographical patterns at the nano-scale mainly induce integrin aggregation, driving the formation of larger focal adhesions, which can affect osteogenic signalling by sequestering focal adhesion kinase [37, 90] (Fig. 3a2). Cytoskeletal contraction is mediated by FAK through Ras homolog gene family member A (RhoA) and extracellular signal-regulated kinase (ERK1/2), leading to activation of runt-related transcription factor 2 (RUNX2), which promotes osteogenesis and new bone formation [91, 92]. A variety of micro- and nanoscale topographies have been applied to bone-regeneration-related biocompatible materials to mimic native micro/nano structures in previous studies. The following part reviews typical topographical modifications to promote osteogenesis and new bone formation in recent years.

### Microstructural topographical patterns

Microstructural topographical patterns can be divided into continuous topography (pots, pillars, tubes, rods, etc.), discontinuous topography (grooves, ridges, fibres, etc.) and three-dimensional (3D) surface topography (surfaces with high curvature, concave areas, etc.) based on approximate topological relationships [37, 93, 94]. These topographies can be realized through various manufacturing methods, including acid-etching, ultraviolet nanoimprint lithography, microparticle suspension, standard soft lithography, and electrospinning techniques.

#### Discontinuous topography

**Fig. 4:**
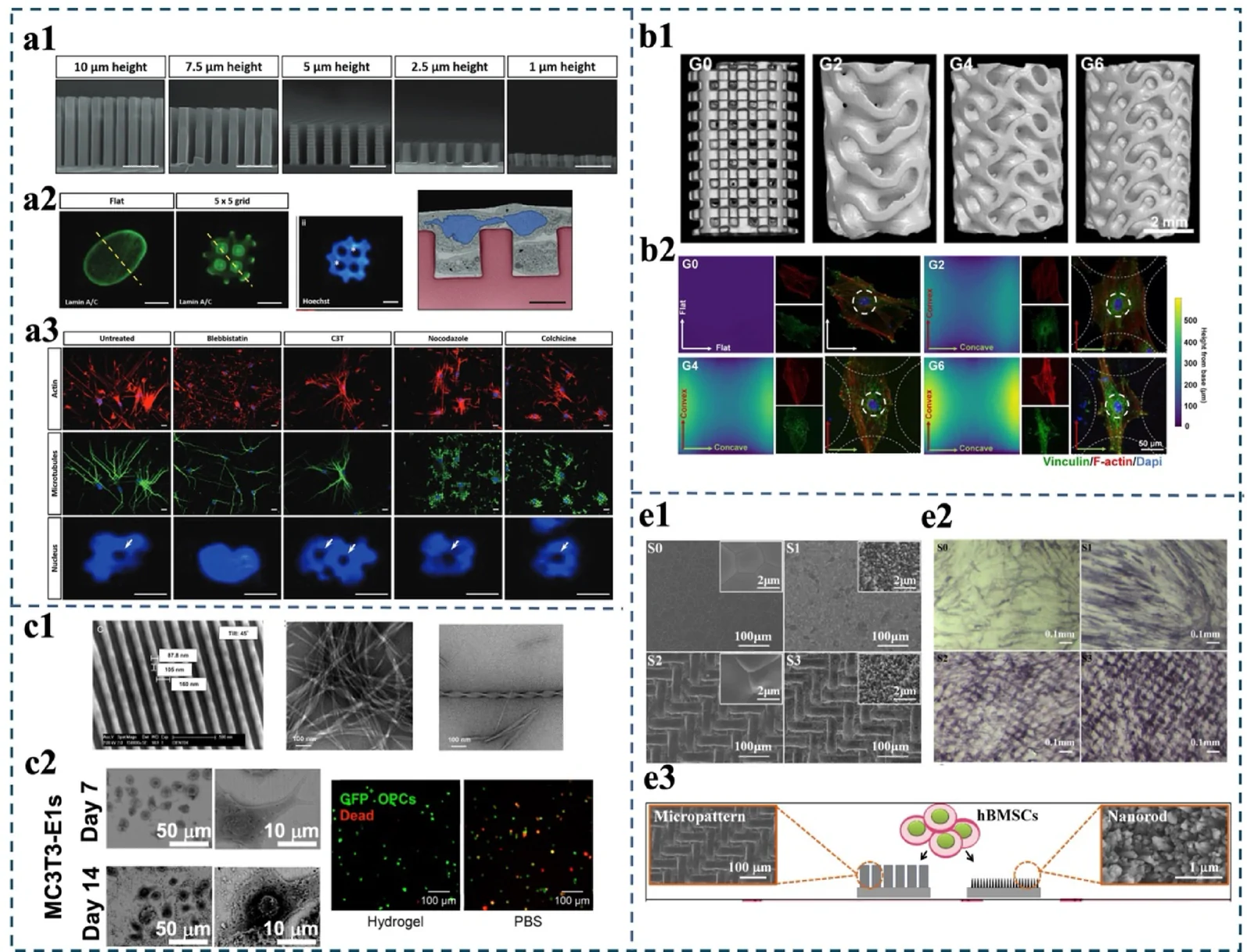
Overview of topography structures and their impact on cell biology.(**a1**) SEM graphs of micropatterned designs [87].(**a2**) Representative images of lamin A/C (green), DNA (blue), and actin (red) and representative false-coloured FIB/SEM image of MSCs cultured on micropillars. Micropillars are shown in red, with nuclei shown in blue [87].(**a3**) Representative fluorescence microscopy images of MSCs treated with actin and microtubule inhibitory drugs—blebbistatin (myosin II inhibitor) and C3T (RhoA inhibitor) or nocodazole (microtubule polymerisation inhibitor) and colchicine (anti-mitotic inhibitor)—during culture on the micropatterned design. Actin is shown in red, microtubules are shown in green, and nuclei are shown in blue. Arrows indicate regions of nuclear indentation and DNA displacement around the micropillars [87].(**b1**) Overview of hyperboloidal topographical scaffolds [101].(**b2**) Immunofluorescence staining of vinculin (green), F-actin (red), and nuclei (blue) in hMSCs on hyperboloidal surfaces, demonstrating cellular deformation [101].(**c1**) SEM images of nanogroove templates and nanofibre structures [133, 134].(**c2**) Cell morphology of MC3T3-E1s on nanogroove structures and cell viability in nanofibre hydrogels [133, 134].(**e1**) Field-emission SEM images of samples with a smooth ceramic surface (**S0**), a nanorod surface structure (**S1**), a micropatterned surface structure (**S2**) and a micropattern/nanorod hybrid surface structure (**S3**). The insets in the top-right corners of S0, S1, S2 and S3 show the corresponding high-magnification images, respectively [145].(**e2**) ALP activity of cells cultured on samples S0–S3 with different surface structures after 14 days [145].(**e3**) Schematic illustration of the micropattern/nanorod hybrid surface structure [145]

Discontinuous topography mainly comprises morphological forms of separated structures, including tubes, pillars, rods, hemispherical shapes, porous shapes, honeycomb-like patterns, and pits. Discontinuous topography has been shown to promote bone regeneration (Fig. 3b). Zhu et al. [95] created micro- and nano-hemispherical topographies on titanium surfaces and found the micro-hemispherical topographical surfaces exhibited better MC3T3-E1 cell adhesion, spreading and proliferation than untreated Ti surfaces [95]. Jiang et al. [96] compared the osteogenic properties of Ti surfaces with different discontinuous topographies. It was found that micro/nanograss Ti, with grass-like randomly arranged short fibres and microscale porous structure, exhibited the better osteogenic properties than micro/nanoclump and micro/nanorod structures in vitro and in vivo, with higher expression of osteogenesis-related and focal-adhesion-related proteins including vinculin, integrin β1, Runx2 and OPN [96]. To clarify the underlying mechanisms of topography on osteogenic differentiation, Carthew et al. [87] developed a micropatterned library consisting of square pillars with heights of 2.5–10 μm and widths of 1 μm. The study highlighted that the mechanotransductory signalling plays an important role in osteogenesis (Fig. 4a1). They demonstrated that changes in nuclear morphology and chromatin architecture, caused by forces from cytoskeletal tension and the micropillar topography, are a major driver of osteogenesis [87] (Fig. 4a2). Carthew’s work has advanced the study of topography for osteogenesis from the cell-shape scale to the nucleus-shape scale, using actin and microtubule inhibitory drugs to illustrate the relationship between morphology and gene expression. Moreover, MSC osteogenesis is enhanced by micropillar designs that alter chromatin organization and gene expression, directly linked to nuclear deformation caused by myosin-II-generated tension on the actin cytoskeleton (Fig. 4a3).

#### Continuous topography

Continuous topography mainly includes continuous and linear topographical surfaces, such as grooves, ridges, and fibres. Microgrooved topological surfaces, due to their linear, aligned and neat features mimicing the unique characteristics of the natural periosteum, which possesses an exquisite topography consisting of longitudinally oriented cells and collagen fibres [97] (Fig. 3b), have the ability to regulate the osteogenesis of cells in a manner similar to the natural periosteum [98, 99]. Shi et al. [100] developed a microgrooved topographical morphology on the surface of nanosheets to replicate the natural periosteum’s ability to guide the direction of bone development. The expression of key osteogenic markers in cultured stem cells, including Runx2, ALP, and osteocalcin, was effectively promoted. This bone-development-guiding effect can also be observed in other discontinuous topographies. Jin et al. [101] fabricated poly(lactate-co-glycolate)/fish collagen/nano-hydroxyapatite fibrous membranes with aligned topologies using the electrospinning technique and observed superior immune cell recruitment and osteogenesis properties in in vivo experiments [101]. Their research revealed the recruitment of macrophages/monocytes and a significant upregulation in the expression level of M2 macrophage marker genes in the osteogenic microenvironment of the microtopographical structures. This confirms that discontinuous topography possesses immunomodulatory ability to improve bone regeneration by regulating the recruitment and polarization of macrophages. Moreover, the upregulation of VEGF and activation of the HIF-1α pathway confirmed the pro-angiogenic effect of discontinuous topographical structures on bone regeneration. This neovascularization provides the oxygen, nutrients and paracrine signals required for osteoprogenitor survival and matrix deposition. The former sets up a pro-osteogenic niche by controlling inflammation and matrix remodelling, and the latter ensures metabolic support and osteo–angiogenic coupling throughout the regenerating tissue.

#### 3D topography

3D topography mainly encompasses specific surface characteristics, including pattern with different Gaussian curvature, such as concave, convex, hyperboloid features and embossments with 3D surface geometry (Fig. 3b). Compared with the high precision required for micro/nanoscale continuous and discontinuous topographies, 3D surface topography has lower requirements for manufacturing precision because its curvatures are much larger than the cell size (ranging from hundreds to thousands of micrometres). On the other hand, 3D surface topography has a larger cell adhesion area, fewer stress concentration and increased mechanical strength, which could enhance cellular adhesion greatly [102, 103] (Fig. 4b1 and b2).

3D surface topography was proven to have a significantly positive osteogenic effect [104]. The underlying mechanisms are closely related to mechanotransduction and nuclear morphology. Surfaces with varying curvatures exert distinct effects. Concave topographies enhance the osteogenic differentiation of MSCs by promoting the expression of bone-regeneration-related proteins, including Runx2, osterix, and osteonectin [105]. This process is mediated by α2 and α5 integrins through mechanotransductory signaling [105]. Additionally, cells on concave surfaces exhibit faster migration speeds due to a reduced contact area [94]. In contrast, convex surfaces exert greater biomechanical forces—such as increased push force, cytoskeletal traction, and hydrostatic pressure—leading to more pronounced nuclear morphological changes, elevated LaminA levels, and stronger osteogenic differentiation compared to concave surfaces [94]. By integrating concave and convex geometries, hyperboloidal topographies induce nuclear deformation and cytoskeletal reorganization to activate integrin-mediated FAK/MAPK pathways, orchestrating osteogenesis-angiogenesis coupling for accelerated bone healing [102]. In addition to hyperboloidal topographies, tunable curvature serves as an effective approach to achieving multi-curvature 3D surfaces. By utilizing shape-memory polymers, such dynamic platforms can mechanically drive nuclear deformation, significantly enhancing stem cell osteogenesis in a curvature-dependent manner [106].

Currently, the specific application of 3D topography with different Gaussian curvature is as the pore morphology of scaffold-based implant materials [102, 107–109]. Because different Gaussian curvatures lead to distinct physical microenvironments around 3D surfaces, coupling fluid mechanics with scaffold geometry enables a more precise description of the local biological environment [108]. Recent studies show that 3D geometry-dependent parameters such as pore shape and curvature regulate wall shear stress and permeability under flow, thereby directing osteogenic behaviors and matrix formation [110, 111]. In particular, optimized distributions of shear stress on the surface of 3D topography can enhance mechanotransduction signaling in osteoprogenitor cells, while excessively high or low shear levels may lower the flow-mediated responses [108]. This integration with multi-physical modalities represents a growing research trend. On the other hand, multicellular coordinated osteogenic regulation has also become an important direction in 3D surface topography research. Distinct Gaussian curvatures can modulate osteogenic cell behaviour while simultaneously promoting skeletal stem cell recruitment, vascular formation and macrophage polarization toward pro-regenerative phenotypes [107, 109].

Collectively, these studies suggest several common design principles. First, microscale discontinuous topographies primarily regulate cell morphology and cytoskeletal tension. Second, osteogenic enhancement depends not only on feature geometry but also on immune and angiogenic modulation. Third, substantial variability exists in feature size and fabrication methods, making direct comparison difficult. Future studies should establish standardized geometrical descriptors and investigate how discontinuous topographies interact with electrical microenvironments.

### Nanostructured topographical patterns

With the rapid advancement of nanofabrication techniques for bioactive materials, nanostructured topographies have been widely studied in recent years, and more evidence is being collected on the importance of nanoscaled structures in directing cell response [29]. Similar to the above, nanostructured topographical patterns can be divided into continuous and discontinuous topographies. Precision manufacturing processes, such as anodizing, ICP-RIE treatment, plasma spraying and colloidal lithography, which are applied in fields such as intelligent communication devices, aerospace engineering and high-precision instrument manufacturing, are widely used in the fabrication of nanostructured topographies.

#### Discontinuous topography

Discontinuous nanostructured topographies mainly include nanoparticles, nanowires, nanotubes, nanopillars and nanorods. Unlike microstructured topographies, which induce osteogenesis by altering cell shape and directing nuclear and chromosomal structural remodelling, nanostructured topographies promote osteogenesis by regulating stem cell fate at a much smaller and finer scale. Cells are several orders of magnitude larger than their surrounding nanoscale topographical features, which means that the changes in cellular shape are remarkably insignificant compared with microstructured topographies, leading to the formation of unbundled actin fibril-driven filopodia, which serve as the initial structures that allow cells to probe the surface and promote osteogenesis [86]. Filopodia can directly induce focal adhesion kinase (FAK) phosphorylation, which is attributed to the nanoscale similarity between the materials and cell receptors, such as integrins [86, 112]. FAK is a key regulator in the assembly of mechanotransduction-related proteins, including paxillin, α-actinin, talin, and vinculin [86, 113]. Recent studies have identified additional crucial mechanotransducers in this network: for instance, Kindlin-2 promotes cytoskeleton assembly via the integrin β1/FAK/RhoA pathway in a YAP-dependent manner [114], while the actin-associated protein XB130 interacts with Src to activate downstream PI3K/Akt/β-catenin signalling upon nanomechanical stimulation [115]. Furthermore, sensing nanotopological cues involves key effectors like PYK2, which plays a pivotal role in maintaining bone homeostasis by simultaneously promoting osteogenesis and inhibiting overactive osteoclastogenesis [116]. Regulation of these cytokines can drive changes in fibroblast and MSC cytoskeletal and nucleoskeletal morphology, with chromosomes repositioning relative to each other as the cytoskeleton and nucleoskeleton rearrange in response to the topography, thereby attributing to osteogenesis [117–119].

Due to the operability and versatility of nanostructured topographies, their functionalities have been vastly expanded to address complex clinical challenges, such as infection, inflammation, and impaired neuroangiogenesis. From a purely physical perspective, high-aspect-ratio nanostructures (e.g., nanopillars or nanowires) can simultaneously promote MSC adhesion while preventing bacterial colonization by physically piercing bacterial cell membranes [90, 120–122]. Concurrently, these specific physical nanosurfaces can regulate macrophage polarization to downregulate proinflammatory cytokines and inhibit osteoclast differentiation [123, 124]. To go a step further, recent research has evolved from static physical topographies to responsive, multifunctional delivery systems. These advanced nanostructures serve as versatile platforms capable of loading and releasing a diverse array of therapeutic agents, including bioactive metal ions(e.g., Sr^2+^, Zn^2+^, Co^2+^/^3+^), antimicrobial proteins(e.g., lactoferrin), osteoangiogenic growth factors(e.g., VEGF), and functional organic molecules(e.g., riboflavin). For instance, ion-doped nanostructures and nanoenzymes can reprogram macrophage metabolism and scavenge ROS to maintain robust anti-inflammatory and neuro-osteogenic effects [125, 126]. Concurrently, advanced hierarchical designs—such as core-shell arrays or “sandwich” nanorods—enable the precise spatiotemporal release of these loaded agents [127]. Collectively, the synergy between topography-induced mechanotransduction and cargo-driven biochemical regulation endows these composite surfaces with robust antimicrobial, immunomodulatory, and pro-angiogenic benefits, successfully rescuing osseointegration even in compromised pathological microenvironments like diabetes or localized infections [127–129].

#### Continuous topography

Continuous nanotopographies mainly encompass nanogrooves, nanoridges and nanofibers. Similar to discontinuous microtopographies, which alter cellular shape and elongation by mimicking the natural periosteal structure, discontinuous nanotopographies can influence filopodial extension and cell guidance, resulting in cytoskeleton alignment and cell rearrangement along the nanoscale guidance cues [98, 99, 130]. This contact guidance effect plays a crucial role in promoting the osteogenic adhesion and chondrogenic/osteogenic differentiation of stem cells. Furthermore, this osteogenic potential is closely related to the size scale of the nanostructures (e.g., groove depth or ridge width), with specific size thresholds existing for optimal cell alignment and induction [131–133]. With advancements in material manufacturing processes, simple physical topographies have gradually evolved towards multidimensional functionalization. The combination of chemical surface treatments, supramolecular peptide self-assembly, or plasma technologies with nanoscale surface morphologies represents the current research trend (Fig. 4c1 and c2) [134, 135]. In addition to their basic structural functions, continuous nanostructure enable the construction of biomimetic delivery systems and electromechanical microenvironments. Functional continuous nanofibers enable the controlled co-delivery of growth factors, drugs, or herbal extracts. This significantly enhances osteoangiogenic coupling and resolves complex interfacial regeneration like tendon-to-bone healing [136–139]. Concurrently, introducing piezoelectric materials or nano-heterojunctions to mimic natural bone’s heterogeneous electrical microenvironment shows remarkable osteogenic potential, which will be discussed in detail in subsequent Sects. [140–142].

Although continuous nanotopographies consistently improve cell alignment and osteogenesis, the reported optimal dimensions vary substantially among studies. This variability likely reflects differences in substrate chemistry, stiffness, protein adsorption, and experimental models rather than purely topographical effects. Future work should decouple these confounding factors to establish generalizable design rules.

### Hierarchical multiscale topographical patterns

It is a scientific consensus that the native extracellular matrix (ECM) is characterized by a hierarchical multiscale architecture—spanning hydroxyapatite (HAp) nanocrystals and nanoscale collagen fibrils with specific orientations to microscale ordered protein fibres—forming a continuous and complex network of biophysical cues [81, 143]. A critical limitation in current tissue engineering research lies in the over-reliance on simplified, single-scale topographical patterns (e.g., nanogrooves, microgrooves, and uniform nanopillars). These designs fail to recapitulate the synergistic nano-to-microscale biophysical cues inherent to the native osteogenic niche, and this lack of structural complexity has emerged as a major barrier to constructing truly functional macroscopic bone tissues [2]. Therefore, mimicking the hierarchical architecture of native bone via multiscale synergistic topographies is essential for advancing bone regenerative materials.

Multiscale hybrid topographies integrate nanoscale and microscale features (in 2D or 3D frameworks) to synergize microscale cell morphological regulation and nanoscale molecular signalling. Common examples of hierarchical topographies include microgrooved surfaces modified with nanobranches or nanorods, as well as 3D micro-wrinkled structures functionalized with oriented nanogrooves [144, 145]. Such designs guide directional cell spreading, regulate cell/nuclear morphology, and enhance osteogenic differentiation—with hierarchical micro/nanoscale titanium surfaces shown to improve bone–implant contact in vivo by modulating MSC osteogenesis and macrophage polarization [146]. Higher-precision processing methods, higher-resolution hierarchical topographies, and finer nanoscale topological control have enabled the customized design of material interfaces and rapid in osseointegration [147, 148].

The synergistic efficacy of multiscale topographies stems from complementary mechanisms. Microscale features guide cell 3D morphology by enabling penetration of cellular contents (nuclei and cytoplasm) into microscale gaps, inducing cytoskeletal tension, nuclear deformation, and altered gene expression [87, 149]. Cell-imprinted substrates confirm that microscale morphological cues are significant for directing stem cell differentiation and transdifferentiation [150, 151]. As a supplement, nanoscale features trigger integrin clustering and conformational changes, activating signalling cascades (e.g., FAK/MAPK) [86, 152]. Emerging evidence highlights multipathway regulation, including Piezo1-mediated calcium influx, which modulates macrophage polarization, and Hippo/YAP-dependent vascular remodelling [153, 154]. Moreover, such YAP mechanotransduction also activates Wnt-mediated ATP production for enhanced cell activity, while targeted nanotopographies trigger Hedgehog/HIF-1α crosstalk to rescue osteoangiogenic coupling in osteoporotic defects [155, 156].

Notably, optimization is not a simple superposition. Some hybrid surfaces (e.g., nanotubes on microgrooved titanium) do not exhibit superior biological performance and suffer from compromised substrate bonding, thereby hindering clinical translation [157]. Additionally, the coordination of isotropic (nanoscale) and anisotropic (microscale) cues in regulating cell responses remains poorly understood [144].

In summary, simplified single-scale topographies are inadequate for mimicking the native osteogenic niche. To fully recapitulate the complexity of native bone, future research must move beyond static, simplified designs towards hierarchical and dynamically tuneable multiscale interfaces. Intelligent optimization (e.g., gradient surfaces combined with statistical learning) is also needed to address structure–function matching limitations [157] to advance bone regenerative biointerface engineering.

## Electrical signal affects bone regeneration

Bioelectricity is an integral part of the organism at the individual level, playing a crucial role in the regulation of the cell cycle, migration, proliferation, and differentiation at the cellular level. Electrical signals and electrical fields are essential for many cellular growth and development processes [160]. Endogenous electric fields, which consist of transmembrane potentials, have been identified in cardiac, nerve and bone tissues, playing a significant role in wound healing and tissue regeneration processes [161]. Bone tissue is composed of calcium phosphate (CaP) nanocrystals wrapped in collagen fibres [162], which are able to slide over each other under mechanical stress, thereby separating positive and negative charges, which in turn produce a piezoelectric effect and generate a physiological potential.

Therefore, the electrophysiological properties of bone tissue can be utilized as a useful tool for enhancing bone-healing ability and promoting tissue regeneration [163]. In recent years, research on the promotion of bone tissue healing and regeneration accelerated by electroactive materials has rapidly increased through the use of biomimetic exogenous electric fields or electrical stimulation [164].

**Fig. 5:**
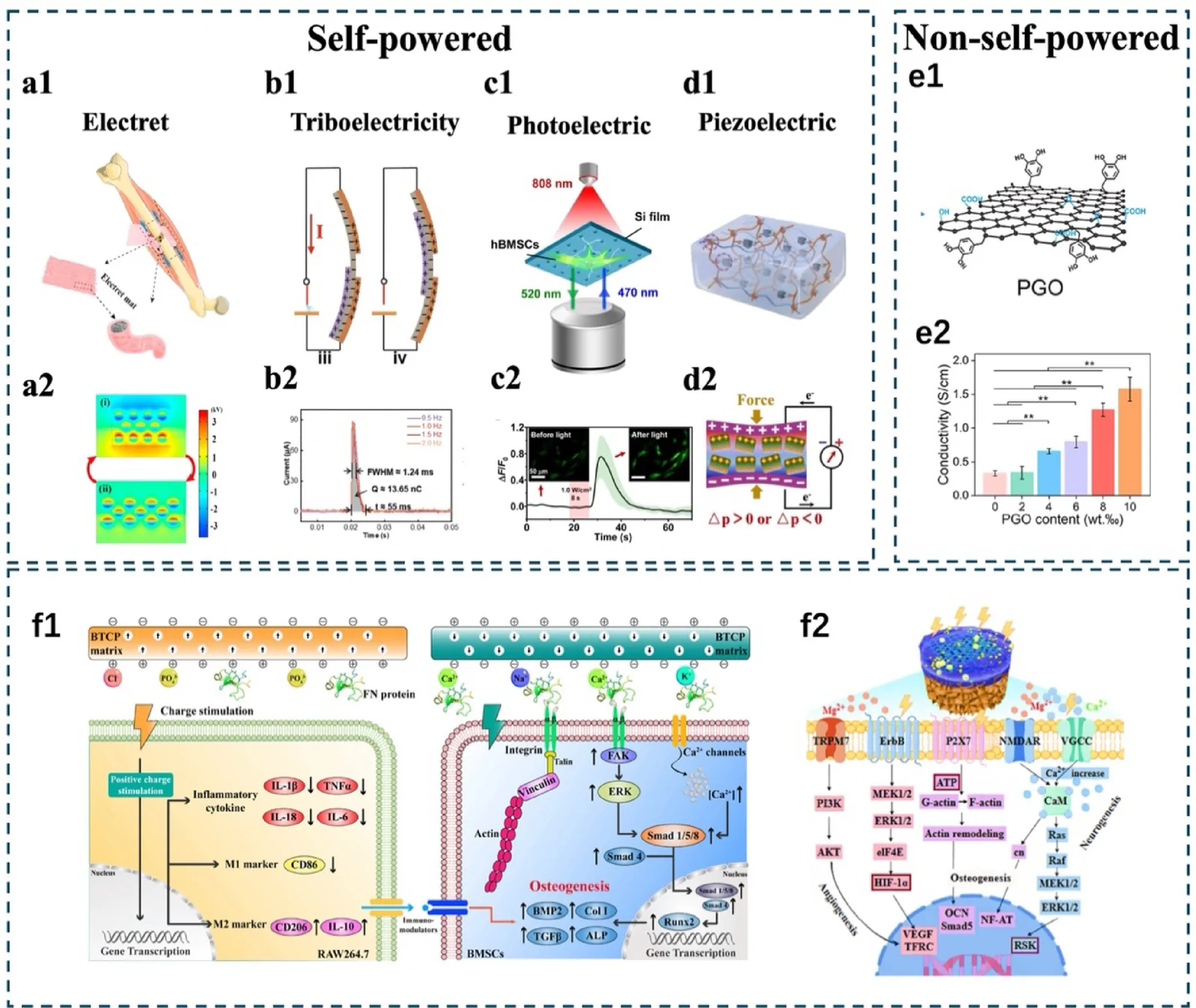
Schematic overview illustrating how self-powered and non-self-powered bioelectric materials affect cell behaviour through modulation of Ca^2+^ concentration and downstream pathways.(**a1**) Schematic diagram of an electret-based nanofibre applied to a bone injury in vivo. [A host-coupling bio-nanogenerator for electrically stimulated osteogenesis](**a2**) Numerical calculations of the potential distribution on the surface of an electret-based nanofibre and within the electret under mechanical interaction. [A host-coupling bio-nanogenerator for electrically stimulated osteogenesis](**b1**) Schematic diagram illustrating the working principle of electric current generation in the triboelectric generator. [Bone Repairment via Mechanosensation of Piezo1 Using Wearable Pulsed Triboelectric Nanogenerator](**b2**) Magnified current peak at vibration frequencies [188].(**c1**) Schematic of photoelectric depolarization for cells [191].(**c2**) Ca2 + signals of a cell in response to illumination under different intensities [191].(**d1**) Schematic diagram of the piezoelectric nanocomposite hydrogel accelerating bone defect healing [249].(**d2**) Schematic diagram of an ultrasound-powered hydrogel generating voltage under pressure [249].(**e1**) Schematic illustration of the conductive scaffold platform [168].(**e2**) Conductivities of conductive scaffolds with different contents [168].(**f1**) Schematic representation of surface charge- and electrical signal-mediated activation of RAW264.7 and BMSC behaviours by electroactive materials [165].(**f2**) Schematic representation of activated pathways mediated by surface charges and electrical signals from electroactive materials across multiple cells in the osteogenic niche [166]

We divide clinically feasible electroactive materials into two categories: self-powered materials and non-self-powered materials. Self-powered materials can be classified into electret, photoelectric, piezoelectric, and triboelectric materials based on the different mechanisms and processes of energy conversion, which allow them to directly transform exogenous stimuli into electrical signals. Bioelectric materials enhance the electricity at target sites, such as neurocytes and stem cells, activating ion channels and pumps on cell membranes, accompanied by changes in the transmembrane potential, thereby guiding a range of cell behaviours and promoting osteogenesis, angiogenesis and neurogenesis [165–167] (Figs. 5f1 and f2). Conductive materials can transmit electrical signals generated by an enhanced endogenous electric field to tissue cells or can be combined with external electrical stimulation to promote cell proliferation and differentiation [168].

### Self-powered materials

#### Electret materials

An electret is a dielectric material possessing a permanent or quasi-permanent electric dipole, serving as the electrostatic equivalent of a magnet. It primarily includes two types: real-charge electrets, formed by injecting and trapping charges in highly insulating dielectrics, and oriented-dipole electrets, created by poling dielectrics above their glass transition temperature and cooling to stabilize the structure [169]. An electret material can be endowed with lasting polarization, inside which the polarized charge of the electret material is retained after being electreted by an external electric field, thereby generating a stable internal potential that delivers intrinsic electrical stimulation [170] (Fig. 5a1 and a2). In biomaterials applications, electret materials are generally classified as either organic or inorganic electrets [171]. Polytetrafluoroethylene (PTFE), polydimethylsiloxane and polyvinylidene fluoride (PVDF) are among the most extensively studied organic electret materials. The electret behaviour of PTFE is generated by negative corona charging, whereby electrons are injected into deep charge traps on both the surface and within the bulk of the polymer, and compensating holes are driven into traps from the reverse side, creating stable space-charge reservoirs that sustain a quasi-permanent internal field [172]. In PVDF, electret functionality is achieved through high-field poling that aligns β-phase molecular dipoles and locks them in place upon cooling, while compensating charges become trapped at crystallite interfaces in deep energy wells, resulting in long-term dipolar polarization and space charge storage [173]. On the other hand, BaTiO3, silicon dioxide (SiO2) and ZnO are widely used as inorganic electret materials. The non-centrosymmetric crystal structure of inorganic electret materials exhibits spontaneous dipolar polarization, producing quasi-permanent internal electric fields that underpin their durable electret functionality [174].

**Fig. 6:**
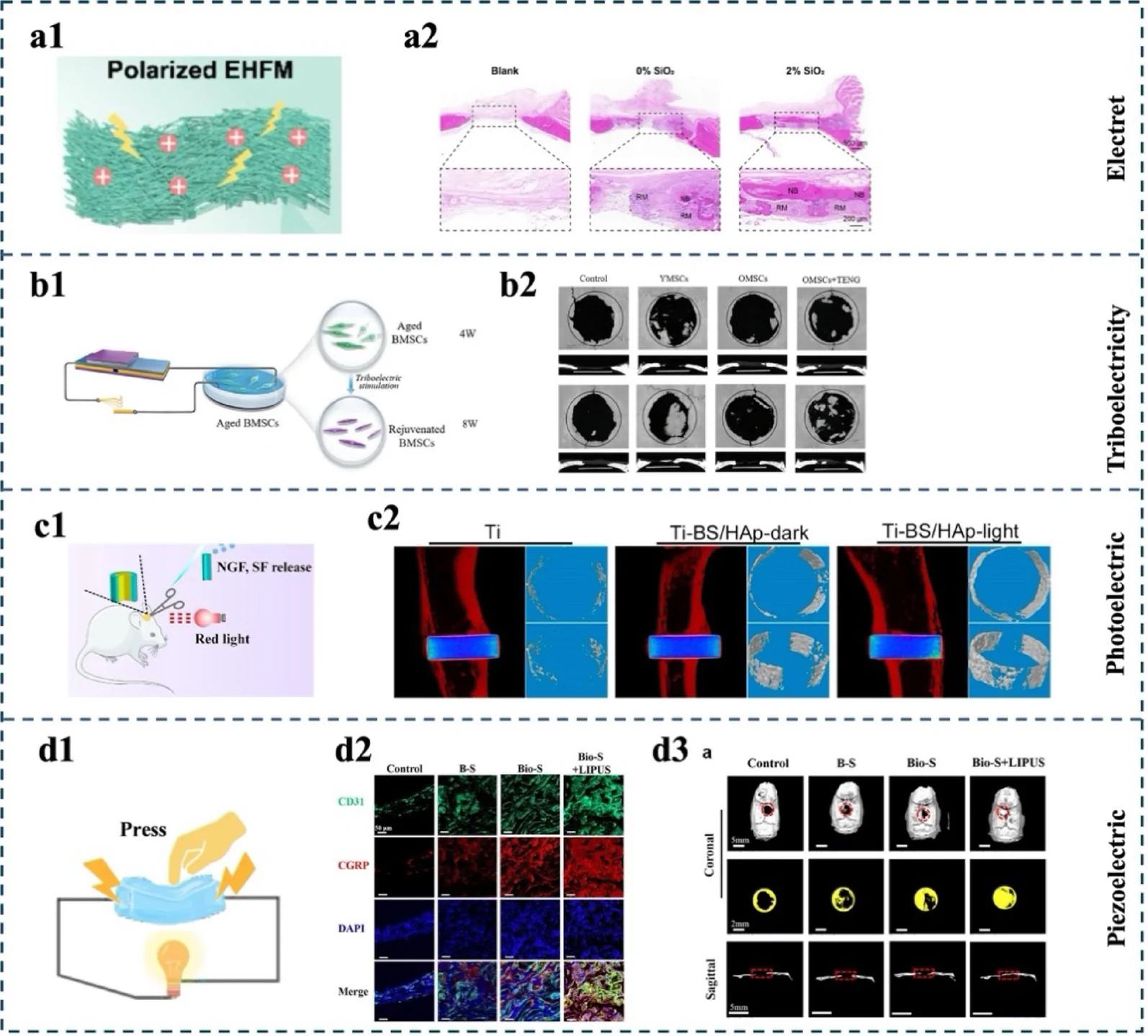
Overview of bioelectric materials and their impact on osteogenesis, angiogenesis and neurogenesis.(**a1**) Schematic diagram of the electret hybrid electrospun fibrous mat [171].(**a2**) Magnified HE staining images of calvarial defects (NB: newly formed bone; RM: residual fibrous mat) [171].(**b1**) Schematic illustration of BMSC rejuvenation via triboelectric stimulation using P-TENG [180].(**b2**) Representative micro-CT coronal and sagittal views of the cranial defect area at 4 and 8 weeks after implantation [167].(**c1**) Schematic diagram of red-light-activated carbon nitride scaffold fabrication [189].(**c2**) Micro-CT 2D and 3D images showing bone formation around Ti, Ti-BS/Hap-dark, and Ti-BS/HAp-light implants. [189].(**d1**) Schematic demonstration of the piezoelectric hydrogel lighting up an LED bulb upon mechanical stimulation [166].(**d2**) Representative immunofluorescence images of CD31 (green) and CGRP (red) in the defect areas after 8 weeks of implantation (scale bar = 5 μm) [166].(**d3**) 3D reconstruction of the coronal and sagittal planes of the skull [166]

In recent years, a common approach to utilizing inorganic electret materials has been the dispersion of particles within bone repair materials. Jinjie Cui et al. developed a core–shell structured electret hybrid electrospun fibrous mat (EFHM) incorporating SiO2 electrets to simulate the extracellular matrix and provide a stable, long-term electrophysiological microenvironment (Fig. 6a1). The EHFM significantly enhanced osteogenic differentiation of BMSCs in vitro and promoted bone regeneration in vivo, achieving bone repair three times greater than the control in a critical-sized rat calvarial defect model (Fig. 6a2). Mechanistically, its effect was linked to activation of the calcium-sensing receptor and elevated intracellular Ca²⁺ levels, offering a promising strategy for clinical bone defect repair [171]. Likewise, Qiao et al. developed a sandwich-structured electroactive membrane by uniformly dispersing SiO₂ electret particles within a poly(dimethylsiloxane) matrix via layer-by-layer blade coating and subsequent polarization. The adjusted surface potential of the composite membrane can promote osteogenic differentiation [170]. In addition, Bandyopadhyay et al. fabricated TiO₂ nanotube electrodes (TNTs) via anodic oxidation and then imparted surface charge through high-voltage thermal polarization to create TiO₂ electrets (TNT-Ps), exhibiting enhanced new bone formation [175].

On the other hand, organic electret materials are commonly utilized as composite fibre mats and membranes [176, 177]. For instance, electret-based host-coupling bio-generators can harvest host biomechanical energy to provide a continuous electrical microenvironment for in situ stimulation therapy, utilizing surrounding tissues and interstitial fluids as integrated electrodes and load circuits to successfully accelerate new bone formation [176].

#### Triboelectric materials

Static electricity generated by friction is very common in life. A triboelectric nanogenerator (TENG) is a basic component that utilizes the coupling of triboelectric and electrostatic induction effects and a thin-layer electrode to achieve the effective output of current, which can generate friction and form an outward electric flow [178, 179] (Fig. 5b1 and b2). Based on output modes, TENGs can be classified into direct current and electric field types, both capable of modulating cell proliferation and tissue regeneration. Previous studies demonstrated that pulsed TENG stimulation rejuvenates aged BMSCs, reduces senescence markers, enhances proliferation and osteogenic differentiation, and accelerates in vivo bone formation(Fig. 6b1 and 6b2) [180].

Although the triboelectric systems demonstrated broad regenerative potential but were limited by structural instability of electrical polarization and non-degradability, often requiring secondary removal [181]. To address these issues, biodegradable and implantable TENG-based devices were developed to provide sustained electrical stimulation under physiological motion and achieve gradual in vivo degradation, thereby improving bone healing outcomes and clinical feasibility [182]. What’s more, more precisely mimicking biomimetic systems has become a direction in current material design. For instance, self-adhesive TENGs utilizing peptide-grafted polymer electrodes have been developed to deliver motion-responsive bionic electrical stimulation that dynamically simulates natural physiological potentials [183]. Biofeedback-driven TENG platforms can harvest intrinsic biomechanical signals to generate bionic electrical cues, inducing osteoblast metabolic reprogramming toward aerobic glycolysis [184]. Biodegradable 3D-printed triboelectric scaffolds further enable electrically enhanced endochondral ossification [185].

In addition, triboelectrically driven nanogenerators have been demonstrated to exhibit multifaceted tissue regenerative capabilities. For instance, triboelectric immunotherapy enables simultaneous antibacterial activity and M1 macrophage polarization with immune memory formation, improving infection clearance and reducing recurrence, while advanced biodegradable TENG platforms can restore the disrupted “mechano-electro-biochemical” cascade and activate Piezo1-mediated Ca²⁺ signaling to synergistically enhance osteogenesis and angiogenesis [183, 186]. Also, the TENG systems enhance sensitivity to mechanical cues and activate mechanotransduction pathways such as Piezo1–Ca²⁺–HIF-1α, thereby promoting osteogenesis, angiogenesis, and even neural regeneration [187, 188].

#### Photoelectric materials

When the frequency of light exceeds a threshold value, a metal surface emits photoelectrons immediately after irradiation, and the photoelectric effect occurs [189]. Materials with photoelectric characteristics can be divided into organic and inorganic materials (Figs. 5c1 and c2). Common inorganic materials include Si and carbon nitride, whereas organic materials include hydrophilic poly(3-hexylthiophene) nanoparticles (P3HT NPs) [190].

Similar to inorganic electret materials, inorganic photoelectric materials are generally used as dispersed reinforcing particles in conjunction with other forms of bone repair materials, such as hydrogels, implants, bioscaffolds and composite membranes. Wang et al. [191] presented a 3D biodegradable optoelectronic bioscaffold for bone tissue regeneration. In response to near-infrared (NIR) illumination, the Si structures generate electrical signals that depolarize cell potentials and induce intracellular calcium activities. Under illumination with an 808 nm laser at an intensity of 200 mW/cm^2^, the Si-based optoelectronic scaffold produces a transient capacitive current with a peak value of 1.6 nA. Experiments showed that photostimulation of Si films could effectively regulate the osteogenic differentiation of hBMSCs and enhance the bone conduction and induction properties of bone regeneration scaffolds. Jieni Fu et al. created a photoelectric-responsive bismuth sulfide/hydroxyapatite (BS/HAp) film, which can activate Na⁺ channels in MSCs, induce membrane depolarization and Ca²⁺ influx, and trigger Wnt/Ca²⁺ signalling to promote osteogenic differentiation under NIR light [167] (Fig. 6c2). Likewise, Anna Papaioannou et al. developed 3D hydrogel scaffolds by using graphitic carbon nitride as a sustainable visible-light photoinitiator to crosslink gelatin methacrylamide and dopamine methacrylamide in the presence of 50 wt% nanohydroxyapatite. This biomimetic composite exhibited enhanced mechanical strength and long-term stability in PBS and supported high pre-osteoblast viability and accelerated differentiation towards mature osteoblasts, demonstrating its potential as a robust, green-chemistry scaffold for bone tissue engineering [192].

The photo-thermo-electric effect represents another way to convert light energy into electrical energy. It is a coupled process where light absorption induces localized heating via non-radiative relaxation to create a temperature gradient. This thermal gradient subsequently drives the directional diffusion of charge carriers through the Seebeck effect, converting light into an electrical potential [193, 194]. For instance, Bi_2_Te_3_/Ti_3_C_2_Tx heterojunctions into polymer scaffolds. Under NIR irradiation, Ti_3_C_2_Tx generates heat to provide a temperature gradient, activating the thermoelectric effect of Bi_2_Te_3_. These heterojunctions enhance carrier mobility and charge separation, producing electrical signals that facilitate Ca²⁺ influx and osteogenesis [195, 196]. Another implementation utilizes NIR-responsive ZnO/black TiO2-x heterojunction nanofilms immobilized on titanium implants. This structure improves interfacial charge transfer, endowing implants with broad-spectrum antibacterial efficacy while promoting macrophage pro-regenerative transformation and osteogenesis via the Wnt/β-catenin pathway [197].

#### Piezoelectric materials

Piezoelectric materials are non-centrosymmetric dielectric materials that generate electric charges on their surfaces under applied mechanical stress and conversely undergo mechanical strain when subjected to an electric field [198]. Traditional electrical therapies utilize direct current or alternating current stimulations for bone regeneration, involving implantable electrodes tethered to control circuits and power supplies [199]. A piezoelectric material is a new type of intelligent material, which induces surface dipole rearrangement under external pressure or direct current input, resulting in an uneven distribution of internal anions and cations through polarization, thereby generating a continuous, uninterrupted potential difference [200] (Fig. 5d1 and d2). Piezoelectric materials generally comprise polymers, ceramics, and composites. Owing to recent research advances, these materials have been utilized in multiple formats for bone regeneration, including coatings on implants, bioactive scaffolds, hydrogels, and composite membranes tailored for bone repair [201]. Piezoelectric materials can be divided into organic and inorganic piezoelectric materials [201].

Organic piezoelectric materials offer high flexibility, good biocompatibility, and environmental friendliness but lacking in piezoelectric coefficients compared to inorganic counterparts [201]. Poly(L-lactic acid) (PLLA), a semi-crystalline polymer containing carbonyl (C = O) dipoles, is a commonly used organic material that relies on pressure to generate a piezoelectric effect. Under the action of stretching or electric polarization, the α crystal form of PLLA may be transformed into the β crystal form. The carbonyl dipoles are arranged along the stretching direction or the electric field direction, thus giving PLLA piezoelectric characteristics [202]. Nathalie Barroca et al. demonstrated that applying mechanical stress to PLLA via an AFM tip induces electrical polarization and modifies surface potential, with the effect varying according to thickness and crystallinity [203]. As a commonly used piezoelectric material, PLLA is frequently fabricated into nanofibers, biomimetic fibrous barrier membranes, and dispersed microspheres for bone defect repair [204–207].

Another organic material that produces piezoelectric effects from carbonyl(C = O) groups is polyhydroxybutyrate (PHB). To improve the electrical and piezoelectric properties of PHB, common measures include doping polyaniline (PANi) [208] and reduced graphene oxide (rGO) [209] into the PHB matrix. After studying composite materials doped with rGO or PANi, Ruo Yu Sheng et al. [210] found that compared with undoped PHB, these materials exhibited a lower water contact angle and higher surface energy. The piezoelectric response and electrochemical impedance are also improved at specific doping ratios.

To overcome the limitations of PHB, such as brittleness, slow degradation and difficult processing, poly(3-hydroxyvalerate) (PHV) was copolymerized to form PHBV films (poly(3-hydroxybutyrate-co-3-hydroxyvalerate). Hiu Fan Liu [211] found that PHBV doped with antioxidized polydopamine-modified hydroxyapatite (PHA) and barium titanate (PBT) showed improved osteogenic differentiation and mechanical properties. Furthermore, PHB has been engineered into microfibrous scaffolds doped with magnetic nanocomposites and piezoelectric nanofiber scaffolds incorporated with barium titanate. These advanced material forms enable synergistic magnetoelectric and piezoelectric stimulation to significantly accelerate bone defect repair [212, 213].

Inorganic piezoelectric materials such as barium titanate and ZnO are widely-used in treatment of bone formation, offering high piezoelectric coefficients but limited in hardness, brittleness, and poor biocompatibility [201]. Therefore, organic-inorganic composite piezoelectric materials currently represent a relatively prevalent application form. For example, barium titanate/PLA Scaffold, ZnO in silk fibroin hydrogels, ZnO/PLAA microfiber, barium titanate/gelatin methacrylate hydrogels, etc. [214–217]. These organic-inorganic composite materials possess not only sufficient piezoelectric properties to deliver the requisite electrical stimulation, but also adequate plasticity and flexibility to conform to bone defects of varying geometries.

Applying voltage to a piezoelectric material can also induce surface dipole rearrangement to produce potential differences. Xiaohan Dai et al. implanted BaTiO3/poly(vinylidene fluoride trifluoroethylene) (BTO/P(VDF-TrFE)), which was polarized using corona discharge under a direct current field of 13 kV to stimulate a biomimetic electrical microenvironment. showing the promoted osteogenesis via the AKT2–IRF5 signalling pathway [218]. Similarly, potassium sodium niobate bioceramics fabricated via high-pressure polarization have demonstrated the capability to regulate the severe inflammatory microenvironment characteristic of osteoporosis [217].

For piezoelectric materials, a force stimulus is required to trigger charge generation; however, long-term external mechanical loading is impractical for clinical use. Therefore, ultrasound-responsive systems have emerged, in which high-energy acoustic waves activate piezoelectric materials to generate electrical output. Low-intensity pulsed ultrasound (LIPUS), as a non-invasive mechanical wave, has been widely demonstrated to promote fracture healing and bone regeneration [219–221]. Beyond single osteogenic regulation, emerging strategies emphasize microenvironment-responsive and disease-specific modulation. By integrating sonosensitizers or targeting membranes, LIPUS can remove drug-resistant biofilms via ROS-induced bacterial ferroptosis and reprogram macrophage metabolism to resolve severe infectious bone defects [222, 223]. Furthermore, LIPUS can achieve synergistic dual-action therapies by coupling piezoelectricity. This allows for the “two-way regulation” of bone homeostasis -- simultaneously promoting osteogenesis and inhibiting osteoclastogenesis in osteoporotic conditions and facilitates the integrated repair of articular cartilage and subchondral bone [224, 225].

In addition to providing energy through external ultrasonic stimulation, microscale cell motility has been experimentally demonstrated to effectively trigger piezoelectric mechanical-to-electrical conversion. In the study by Zhirong Liu et al., rBMSCs were cultured on PVDF nanofibres. They found that the traction forces exerted by the cells induced microscale deformations of the nanofibres, thereby eliciting piezoelectric responses that generated Ca²⁺ flows. These Ca²⁺ dynamics enhanced expression of neuron-specific markers and promoted neuronal differentiation [226]. Correspondingly, Wang Yanlan et al. reported that migration-associated forces in adherent cells produced on-demand piezoelectric potentials, which provoked intracellular Ca²⁺ oscillations and activated the Cav3.2-mediated Ca²⁺/calcineurin/NFAT1 signalling cascade [227]. This microscale integration of cellular mechanics and material piezoelectricity points towards an advanced paradigm in biomaterial design.

Moreover, studies have made further progress in elucidating the multicellular impacts of piezoelectric stimulation materials within the osteogenic niche. Liangyu Wang et al. [228] achieved multidirectional tissue-driven osteogenic regeneration through the synergistic coupling of ionic release and electrical stimulation. They developed a piezoelectric whitlockite (PWH) scaffold fabricated from poly(ε-caprolactone), which synergistically integrates Mg²⁺ release and electroactive properties to drive neuro-vascularized osteogenesis. Concurrently, the scaffold’s piezoelectricity, derived from annealed PWH nanoparticles, mimics endogenous bioelectrical microenvironments, promoting neuronal excitability and osteoblast activation. Angiogenesis is augmented through upregulated expression of Ang-1 and VEGF, facilitated by Mg²⁺-mediated endothelial cell recruitment and piezoelectric-driven electrical cues. The Mg²⁺/piezoelectric synergistic integration mechanism of PWH significantly enhances vascularization, neural generation and bone matrix deposition, achieving integrated in situ regeneration of neuro-vascularized bone tissue. Zhao Yao et al. developed a bioinspired bilayer scaffold to mimic the periosteum and bone matrix, which was composed of chelated alginate, gelatin methacrylate, and sintered whitlockite nanoparticles to replicate viscoelasticity and piezoelectricity (Fig. 6d1). The scaffold significantly enhanced osteogenesis, angiogenesis, and neurogenesis when combined with low-intensity pulsed ultrasound-assisted piezoelectric stimulation [166] (Figs. 6d2 and d3).

### Non-self-powered potential material

#### Conductive Materials

Conductive materials are substances capable of transmitting electrical signals generated by an augmented endogenous electric field to tissue cells and can also be integrated with external electrical stimulation [229–232]. Compared with non-conductive materials, conductive materials significantly promote cell adhesion and accelerate cell migration, proliferation and differentiation, thereby achieving enhanced protein secretion at the material–tissue interface and facilitating ideal osseointegration [233]. There are many types of conductive materials, among which graphene oxide (GO) and polypyrrole (PPy) have garnered increased attention in recent years due to extensive research.

Graphene (GP) is an excellent conductive material with few impurities [234], low density, strong ductility, and high electrical conductivity. Mussel-inspired polydopamine (PDA) is considered to be a promising antioxidant that reduces oxidative stress by scavenging ROS and down-regulating inflammatory mediators [235]. Yazhen Li et al. [168] designed a scaffold consisting of PDA-reduced graphene oxide (PGO) and PDA-modified hydroxyapatite nanoparticles (PHA), dispersed within an alginate and gelatin (AG) network to form an immunomodulatory scaffold for periodontal bone regeneration in diabetes (Fig. 5e1 and e2). The PDA modification allows the PGO to be evenly dispersed in the scaffold, thus providing a well-connected conductive path. At the same time, catechol groups on the scaffold promote cell adhesion while also conferring effective reactive oxygen species scavenging and anti-inflammatory properties. When no external electrical stimulation was applied, ALP activity in BMSCs was higher than that in the control scaffold, indicating enhanced osteogenesis. When external ES was applied, the osteogenic effect was gradually strengthened, maximizing at 300 mV.

Graphene–cellulosic (G–C) [236] paper is also an excellent conducting material for the large-scale production of biomaterials. Jianfeng Li et al. [237] uniformly deposited graphene onto a cellulose substrate, which showed porous features decorated through the structure, thereby achieving lower impedance and higher charge injection capacity compared with an Au electrode. Under stimulation at 1 V/cm with a current density of approximately 300 µA/cm2, the transmembrane potential of the cells was altered, thereby promoting cell proliferation and osteogenic differentiation.

To avoid the inconvenience associated with external electronic devices, a functional material that can actively couple endogenous EFs with wound repair is desirable. The transmembrane potentials of many cells are generated and maintained by ion channels and ion pumps [238], which may become leaky following tissue damage. Growth factors also play an important role in tissue regeneration [239]. It has been shown that the independent and direct use of exogenous growth factors is an effective means to induce cellular healing and regeneration. Wang et al. [240] designed a 3D NS-rG@VEGF scaffold. They used nanofibres (NFs) and 3D-printed scaffolds to simulate the extracellular matrix, incorporating reduced oxide graphene (rG) and VEGF. The scaffold exhibits enhanced electrical conductivity and water absorption, enabling early collection of secretions, transmission of bioelectrical signals, and proactive coordination with tissue repair cascades for precise tissue remodelling.

Some obstacles exist in the application of carbon nanomaterials in biomedicine. The high specific surface area makes uniform distribution within a polymer matrix difficult [241], while their high chemical stability renders them resistant to degradation [242], which may result in biotoxicity in the human body. Conductive polymers generally refer to organic polymers that intrinsically possess the ability to delocalize electrons along their backbones when an electrical potential is applied [243]. Conducting polymers with conjugated pi-bond systems along their backbones form extended chains of loosely bound electrons [244]. Upon reduction or oxidation by a dopant molecule, this system allows the polymers to exhibit high electron mobility. In the context of specific bone defects, 3D printing technology has gradually attracted attention in this field. Sayan Deb Dutta et al. [245] studied a highly stable and conductive bioink based on polypyrrole-grafted gelatin methacryloyl (GelMA-PPy), employing a triple cross-linking strategy (thermo-photo-ionically) for direct ink writing-based 3D printing applications. The results showed that the hydrogel ink can achieve high line-of-sight fidelity while exhibiting the unique transcriptional characteristics of the natural bone matrix; in vitro microcurrents can promote osteogenesis. This creates a new approach for bone tissue-related therapy.

In addition, a new class of materials has been developed based on a combination of organic and inorganic conductive materials. As mentioned above, Ppy is a stable material that can retain conductivity for a long time. Graphene oxide is produced through strong oxidant treatment and contains abundant oxygen-containing functional groups, such as –COOH and –OH, which confer increased hydrophilicity and improved dispersibility [246]. It is known to exhibit weak adsorption of nucleic acids [247]. Ribonucleic acid (RNA) plays an essential role in protein synthesis and cellular pathways. Thus, Huijung Kim et al. [248] used a Ppy–GO hybrid film to deliver electrical stimulation and load mRNA, enhancing the osteogenic differentiation of hADMSCs. The results confirmed that GO-mediated RNA protection, together with electrical stimulation facilitating mRNA penetration across the cell membrane, contributes to the cellular uptake of mRNA.

## Surface topography and electrical stimulation jointly affect bone regeneration

Single biomimicking factors such as surface topography and electrical stimulation have been extensively and intensively studied, which dramatically improve osteogenesis and angiogenesis [250]. Electrical stimulation restores the electrophysiological microenvironment by transmitting electrical signals and providing electromechanical and electrochemical cues. The physical connection between topography and electric properties indicates the potential of their combined application for tissue engineering. Thus far, the development of biomaterials with interactive topography and electrical cues still poses a formidable challenge. Relevant research reports regarding the combined application in tissue engineering are limited. Furthermore, most studies focus on nerve regeneration, which has already been reviewed [37] and remains an active area of research. It has also been reported that synergistic stimulation of laser-textured or aligned surface topography and an electric field enhances myogenic maturation of human myogenic precursor cells (hMPCs) and promotes muscle regeneration [251, 252]. More recently, the synergistic cues of topography and an electric field have become a focus in osteogenic niche engineering, acting either through parallel effects or interactive biophysical mechanisms. The underlying biophysical mechanisms of the combined application of topographic modulation and electrical stimulation will be systematically elucidated, along with their integrated practical implementation in osteogenic niche engineering. Although substantial evidence supports the independent osteogenic effects of topographical and electrical cues, the evidence base for their integrated application remains comparatively limited. To facilitate interpretation of the current literature, the level of evidence supporting different biointerfacial strategies is summarized in Table 1.

**Table 1:** Current evidence, key challenges, and future design opportunities for biointerfacial modulation in bone regeneration

Strategy	Evidence level	Main support	Current limitation
Topography	Established	In vitro, in vivo	Parameter standardization
Electrical stimulation	Established	In vitro, in vivo	Dose optimization
Topography, electrical	Emerging	Mainly preclinical	Limited mechanistic understanding
Self-powered systems	Preliminary	Small animal studies	Long-term stability
Multifunctional interfaces	Early-stage	Proof-of-concept	Translation

### Synergistic Biophysical mechanisms of topography and electrical stimulation in bone repair

Emerging evidence reveals that topography and electrical stimulation exhibit interconnected biophysical mechanisms in regulating osteogenesis. On the one hand, engineered topography of electrically conductive substrates or coatings directly optimizes current distribution and remodel electrical fields [253]. On the other hand, specific multiscale surface topography induces the formation of focal adhesions and cytoplasmic skeleton reorganization, which drives faster osteogenesis through mechanotransduction [91, 92]. In this way, the interconnected biophysical mechanisms between topography and electrical stimulation can partially be interpreted as the interactive mechanisms between mechanical stress and electrical fields. Research has demonstrated that cells exhibit a dynamic interplay between mechanical forces and electric fields [254]. This electromechanical coupling can arise through several distinct physical mechanisms, including: (1) piezoelectricity effects, (2) Maxwell stress or electrostriction, (3) flexoelectricity, and (4) ionic diffusion.

**Fig. 7:**
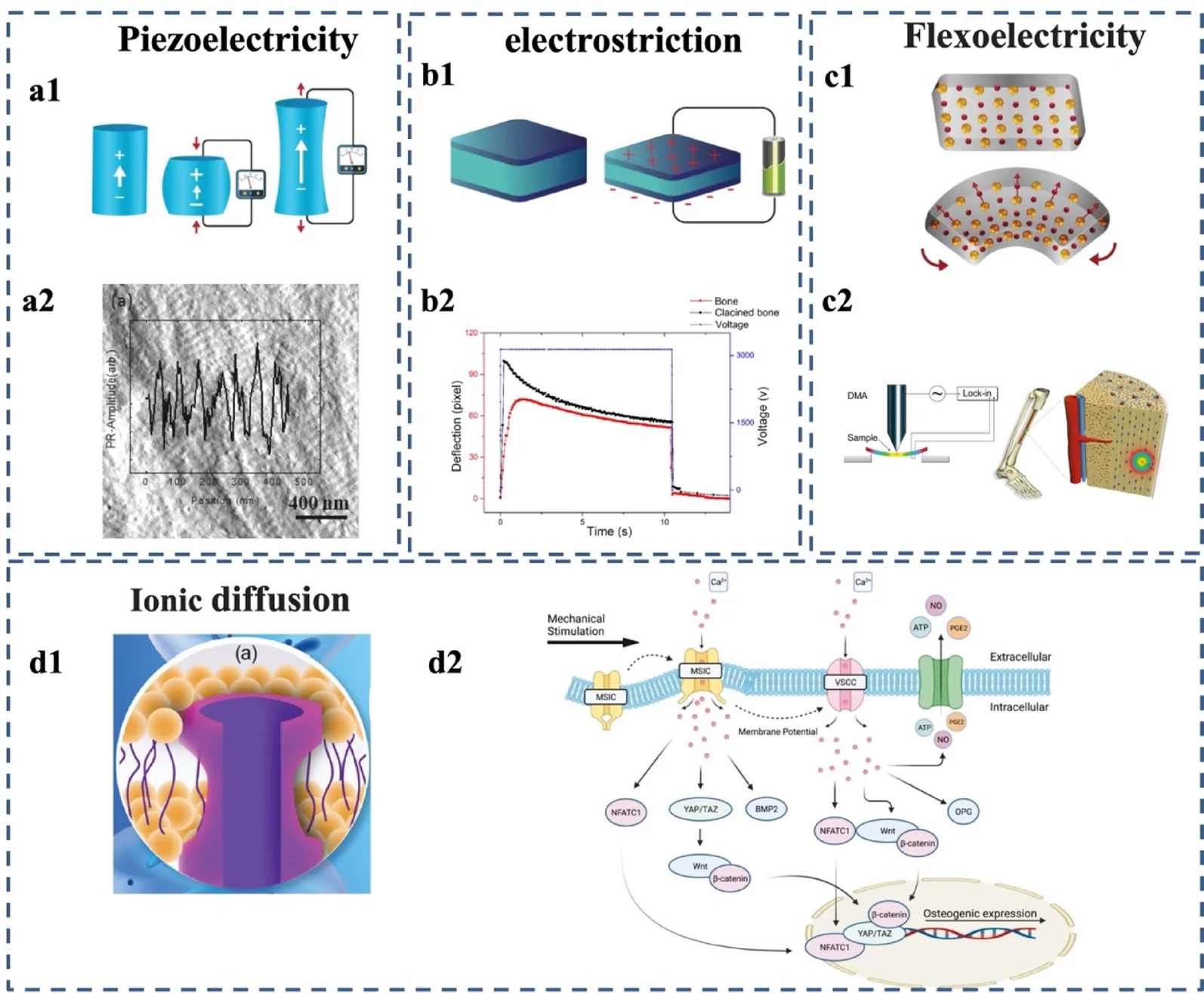
Various mechanisms of electromechanical coupling in bone tissue and cells.(**a1**) Piezoelectricity. A direct linear coupling between uniform mechanical strain (or stress) and electrical field (or polarization), typically observed only in certain crystalline dielectrics that lack centrosymmetry [254].(**b1**) Maxwell stress or electrostriction. A universal phenomenon present in all materials, referring to deformation caused by electrostatic forces developed on opposing electrodes under the application of an electric field [254].(**c1**) Flexoelectricity. A universal phenomenon present in all dielectrics, referring to the notion that nonuniform deformation (such as bending) can induce electrical fields in any material via atomic rearrangement and consequent charge distribution (even in materials that are nominally nonpiezoelectric) [254].(**d1**) Ionic diffusion. Under the action of an electrical field, ions preferentially migrate towards one of the electrodes, thus causing a time-dependent deformation [254].(**a2**) High-resolution shear piezoresponse in cortical bone. a AFM deflection image and b the simultaneously acquired lateral PFM response image showing the shear-piezoelectric response in bone with spatial resolution down to the subfibrillar level of individual collagen fibrils. Inset in a is a line showing the periodic modulation in shear piezoresponse with a periodicity of 60–70 nm [255].(**b2**) Time responses of bending deflections of a bone cantilever in an external electric field. Deflections versus time for bone samples with and without collagens [257].(**c2**) Left: Schematic of the experimental setup for measuring flexoelecricity: a DMA is used to induce three-point bending, and a lock-in amplifier collects the bending-induced current in bone and hydroxyapatite samples. Right: Strain gradients, and hence flexoelectricity, are greatest around small structural singularities, such as fracture fronts (crack tips), in bone mineral [264].(**d2**) Calcium channels and downstream elements. Mechanical stimulation alters the conformation of mechanosensitive ion channels (MSICs), allowing Ca2 + entry. As Ca2 + enters the cell, the change in membrane potential induces activation of voltage-sensitive calcium channels (VSCCs). The influx of Ca2 + is thus utilized by several targets, including NFATC1, YAP/TAZ, canonical Wnt, BMP2, and OPG. This influx can also induce osteogenic transcription [265]

Piezoelectricity refers to the linear generation of electrical polarization in a material with a non-centrosymmetric crystal structure under uniform mechanical stress, and conversely, mechanical deformation in response to an applied electric field [198] (Fig. 7a1). This phenomenon occurs only in anisotropic crystalline dielectrics, typically rigid ceramics. Although most biological cells lack the crystalline structure required for conventional piezoelectricity, shear piezoelectricity has been experimentally confirmed in cortical bone, where collagen fibrils produce a measurable response under lateral piezoresponse force microscopy (Fig. 7a2) [255]. Apart from natural bone tissue or cells, piezoelectricity can also arise at the interface between cells and piezoelectric materials. When cells adhere on flexible piezoelectric materials, the cellular traction force can contribute to the deformation of the material and trigger electrical stimulation. An electroactive membrane was engineered containing piezoelectric barium titanate particles. It was found that during the proliferative and modelling phases, forces generated by adherent cell migration produced on-demand piezopotential and promoted osteogenesis of periodontal ligament stem cells (PDLSCs) via activation of the Ca^2+^/CaN/NFAT signalling pathway [227]. As a surface traction, it directly induces cellular/microenvironmental morphological changes, modulating cell spreading, differentiation, and fate determination—acting as a critical biophysical bridge for electro-mechanical coupling in osteogenic niches. Notably, it is universal in dielectrics and independent of electrostrictive properties.

Electrostriction is a universal bulk electromechanical phenomenon in all dielectrics, where applied electric fields induce internal ion/dipole polarization and lattice/molecular rearrangement, leading to macroscopic deformation [256] (Fig. 7b1). Notably, significant electrostrictive deformation under practically feasible electric fields is predominantly observed in soft dielectrics (e.g., elastomers and biological tissues) and certain biomaterials due to their high compliance—even bone cantilevers have been shown to exhibit this effect (Fig. 7b2) [257]. In contrast, Maxwell stress is a surface-specific effect from electrostatic fields, arising from interface charge accumulation caused by electric field discontinuities (e.g., scaffold–biological environment interface) [258]. Its physical nature is rooted in electrostatic forces acting at material surfaces or interfaces, where discontinuities in the electric field are present. Maxwell stress generated by external electric fields acts as a vital biophysical bridge for electro-mechanical coupling at the cellular level. For further discussions on the difference between the physical origins of electrostriction and the Maxwell stress effect, interested readers are referred to seminal works by Tian et al. [259]. Overall, these field-induced stresses induce morphological deformations and serve as direct mechanical stimuli, effectively modulating cellular behaviours such as spreading and differentiation to direct cell fate [260].

Flexoelectricity, similar to electrostriction but distinct from piezoelectricity, is a universal electromechanical coupling between strain gradients and electric polarization, allowed by symmetry in all materials [261, 262] (Fig. 7c1). This phenomenon holds particular significance in biological systems, especially at cellular scales. In biological membranes, flexoelectricity manifests as dipole moment alterations induced by curvature changes—an efficient transduction mechanism given the membrane’s natural flexibility. It is evidenced by numerous studies that flexoelectricity induced healing of microcracks subject to mechanical loading in cortical bone (Fig. 7c2) [263, 264].

Ionic diffusion plays a crucial role in regulating cellular function. While ions cannot passively diffuse through the impermeable lipid bilayer, specialized transmembrane proteins create selective channels that enable controlled ion passage. These protein channels facilitate ionic movement that simple diffusion driven by concentration gradients cannot achieve, ensuring precise regulation of cellular electrochemical balance (Fig. 7d1). Mechanical deformation is well known to alter the electrochemical potential and thus can directly impact the diffusion process. Osteocytes are believed to be the mechanosensory cells of bone, transmitting mechanical signals to osteoblasts and osteoclasts. Ionic channels, especially potassium and calcium ion channels, are considered as major intercellular communication means for biochemical transmission of a mechanical stimulus in bone cells (Fig. 7d2) [265]. Ionic diffusion induces ion flux in bone cells, resulting in a change in the cell membrane potential, which may be positive (depolarization) or negative (hyperpolarization). The bone cell can quickly identify the characteristics of mechanical stimuli and respond electrophysiologically in different ways, with varying degrees of ion channel activation, resulting in the hyperpolarization or depolarization of the plasma membrane [266]. Conversely, diffusion of ions driven by electrical fields can cause a time-dependent deformation owing to the fact that ions migrate preferentially to one of the electrodes. Because of this mechanism, a thin film containing mobile ions will typically bend under the influence of an electrical field.

Collectively, the four principal mechanoelectrical coupling mechanisms orchestrate bone repair and remodelling processes at cellular and/or tissue scales through tightly coordinated biochemical and biophysical crosstalk.

### Integrated application of topography and electrical stimulation in osteogenic niche engineering

Materials for topography–electrical co-stimulation in osteogenic niche engineering can be classified as topography–external electrical stimulation or topography–surface electrical stimulation materials, depending on how the potential is applied.

#### Topography–external electrical stimulation materials

Topography–external electrical stimulation refers to cultured cells and materials exposed to an electric field applied externally, for example, via transformer-like coupling, pulsed electromagnetic field systems, or multichannel electrical stimulators [66, 267–269]. External electrical stimulation is mainly used in fundamental studies investigating the mechanisms underlying topography–external co-stimulation, rather than in clinical research, due to the practical limitations of applying the electric field outside the petri dish.

Batur Ercan et al. [267] explored the efficacy of electrical stimulation in promoting osteoblast density on nanotube-anodized titanium. Cells seeded on nanotube-anodized titanium were stimulated with constant bipolar pulses, resulting in increased osteoblast density, thereby indicating enhanced osteoblast proliferation [267]. Greeshma Thrivikraman et al. [66] illustrated the interplay of topography and electrical stimulation at the gene and mRNA levels. Cells seeded on the fibre-like surface with electrical stimulation showed intensified mRNA expression levels of ALP and OCN [66]. A more in-depth study by Poh Soo Lee et al. [268] combined continuous nanoparticle topography with external electric field stimulation, demonstrating both the individual and synergistic effects of nanoparticles and electric field stimulation on osteogenic differentiation at multiple levels. Regarding the effect on the material itself, electric field stimulation influences pro-osteogenic factors, including ion release behaviour, coating surface topography, and coating stiffness. Regarding the effect of stimulation on cell behaviour, the up-regulation of osteogenic gene markers, including Runx2/OCN gene expression, and the Wnt/β-Catenin signalling pathway under nanoparticle–electric field stimulation is similar to the continuous processes that occur in vivo to maintain bone homeostasis [268].

As mentioned above, some mechanisms underlying the synergetic effects of topography and electrical stimulation have been well demonstrated in studies on topography–external electrical stimulation materials in recent years, providing theoretical support for subsequent research on topography–surface electrical stimulation materials.

#### Topography–built in electrical stimulation materials

Unlike topography–external electrical stimulation materials, topography–surface electrical stimulation materials generate voltage through the surface potential of the material itself, rather than from an external source. The generation of an electrical field on the material surface, achieved through increased electronic conductivity, piezoelectricity and heterojunctions, can provide a constant, sufficient and noninvasive potential to enhance osteogenic differentiation [270, 271]. Research on the combination of topography and the surface bone-mimetic microdomain electrostatic field serves as a catalyst for studying the mechanisms of topography–external co-stimulation.

Topography–conductive materials: With the implantation of conductive materials, electrical signals generated by endogenous electric fields can be strengthened and efficiently transduced to cells [202]. Combining the enhanced conductivity of the material with surface topography is a viable approach. Bhattarai et al. [272] developed titanium oxide nanotubes coated with polyaniline, showing improved biocompatibility, thermal stability, electrical conductivity, biomineralization and hydrophilicity. The nanotube/polyaniline material provided an effective platform to enhance the attachment, development and proliferation of preosteoblast cells, whose morphology adapts to the surrounding topography. Osteogenic-related markers (such as increased ALP levels and collagen type I secretion) were also promoted [272], opening a new avenue in bone tissue engineering through the combination with nanotube topography.

While such electroconductive platforms primarily amplify osteogenesis through physicochemical cues, emerging strategies now integrate neurogenic modulation to achieve innervated bone regeneration—a critical yet underexplored dimension in functional bone repair. Yanlin Su et al. [273] developed a core–polycaprolactone/shell–DNM biomimetic periosteum (PD) loaded with 3D black phosphorus (BP), creating an electrically active biomimetic periosteum (PD@BP). The PD membrane comprised a coaxial shell–core structure, generating a consistent fibre-direction topography that promoted nerve axonal growth. The anisotropy of the BP membrane endows it with excellent electrical conductivity, allowing the acceptance of upstream electrical signals to stimulate downstream cells and affect cell fate. The results confirmed that the PD@BP biomimetic periosteum enhances the axon initial segment of sensory neurons, regulates dense core vesicle transport, and releases neurotransmitters, thereby promoting the osteogenic transformation of bone marrow mesenchymal stem cells—a process referred to as neurogenic osteogenesis. RNA-seq analysis further confirmed that the biomimetic periosteum mainly induces neurogenesis and osteogenesis via the Fanconi anaemia pathway. This work provides strong evidence that the surface topography–electrical stimulation coupling environment can facilitate intercellular communication between Schwann cells and BMSCs through neurotransmitters.

Topography–piezoelectric materials: Piezoelectric ceramic materials are capable of generating build-in electrical cues in response to mechanical deformation without the need for an external stimulation device [274]. The electrically charged surfaces of piezoelectric materials result from the rearrangements of dipoles that are produced when an external force or a direct current electrical energy is applied, which is called “polorization” [202, 275, 276]. Barium titanate has long been one of the most commonly used materials in piezoelectric biomaterial research. Its synthesized forms, such as nanoparticles and micropillars, are utilized to provide both specific surface topography and electrical signal stimulation [253, 277–279]. Attempts have been made in the exploration of the combined effect of topography-induced mechanical force and electric activation based on piezoelectric materials [280]. Piezoelectric BaTiO_3_ nanorod arrays were constructed on the surface of titanium implants with switchable dynamic and static electrical stimulation. It was found that nanotopography improves the piezoelectric properties of BaTiO_3_ due to decreased Young’s modulus. In addition, the nanorod array combined with static electrical stimulation significantly enhanced the expression of osteogenesis-related genes. Recent research on the mechanism has further shown that nanotopography-mediated mechanical stress dominantly boosted osteogenesis and angiogenesis via Piezo2/Piezo1/Ca²⁺ signalling. Furthermore, enhanced electric activation improved mechanical-stress-mediated osteogenic/angiogenic responses via the PI3K/AKT/mTOR/GSK3β−1 pathway [281]. Based on a similar BaTiO_3_ nano array, Wu et al. [282] engineered a mechanical–electrical coupling microenvironment for innervated bone regeneration. The underlying mechanism was explored via high-throughput sequencing biotechnology. The transcriptomic analysis results indicated that this coupled microenvironment activated Piezo2/VGCC ion channels, enhancing calcium influx in DRG neurons. This triggered PI3K-AKT/RAS pathways, upregulating CGRP release in sensory nerves to promote BMSC osteogenic differentiation.

Apart from bone implants, topography–piezoelectric materials have also been engineered and applied in bone scaffolds to promote vascularization-driven bone regeneration. Wenwen Liu et al. [283] developed a bioactive composite scaffold by coating porous Ti6Al4V with BaTiO_3_ nanoparticles. The piezoelectricity provided a sustainable electrical stimulation to promote osteoconductive and osteoinductive properties through the calcium signalling pathway. The nanoparticles provided a suitable surface topography to increase cellular proliferation and migration due to increased roughness and hydrophilicity. Also, the results revealed that the appropriate physical electrical microenvironment can increase the ability of HUVECs to secrete PDGF-BB and enhance the formation of PDGF-BB/PDGFR-β and downstream molecules through an autocrine pathway to promote vascularization in bone tissue, thereby enhancing vascularized osteogenesis.

**Fig. 8:**
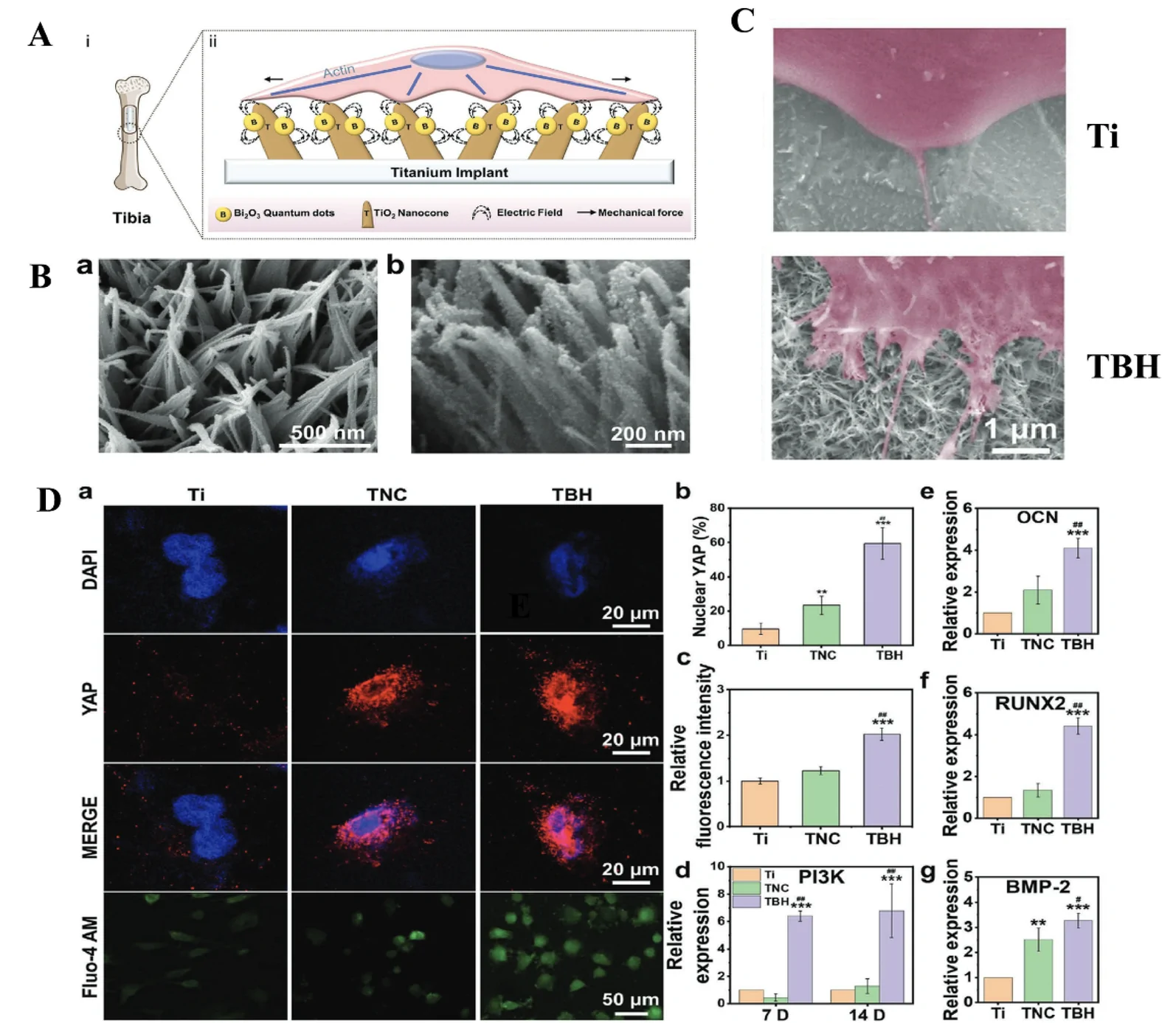
**A**) Schematic illustration of the mechanism by which titanium dioxide nanocone/bismuth dioxide nanodot heterojunctions generate electro-mechanobiological coupling to promote osteogenic differentiation and bone formation. **B**) Characterization of the nano-heterojunction. **C**) SEM images of BMSC adhesion on Ti and TBH surfaces. **D**) Evaluation of the electro-mechanobiological signalling pathway in BMSCs cultured on TBH: **a**) Immunofluorescence images of cells cultured on TBH and controls: DAPI (blue), YAP (red), and Fluo-4 AM (green). **b**) Quantitative analysis of YAP nuclear translocation (*n* = 5). **c**) Quantitative analysis of intracellular Ca2 + fluorescence intensity of TBH and controls. **d**) PI3K gene expression in cells on day 7 and day 14, respectively. These results demonstrate that TBH activates YAP and PI3K cellular pathways. **e–g**) RT-PCR analysis of osteogenic gene markers (RUNX2, BMP-2, and OCN, normalized to GAPDH) on day 7. These results indicate that TBH, providing electro-mechanobiology coupling cues, promotes osteogenic differentiation of BMSCs [270]

Topography–heterojunction materials: Heterojunctions have been widely used in energy conversion, environmental remediation, and photodetection but have not been fully explored in regenerative medicine. Recently, nano-heterojunctions have emerged as a novel platform for engineering a mechanical–electrical environment to support multifunctional bone regeneration. A heterojunction is an interface between two layers or regions of dissimilar semiconductors, where a persistent electric field is formed, providing sustainable and constant electrical stimulation [284]. Huang et al. [270] first reported the in situ fabrication of titanium dioxide nanocone/bismuth oxide nanodot heterojunctions on bone implant surfaces to electro-biomechanically promote osseointegration at the bone/implant interface. They showed that the osteogenic effects of the nano-heterojunctions are mediated by the Yes-associated protein biomechanical signalling pathway and intracellularly induced Phosphatidylinositol 3-kinase signalling pathway. The hierarchical multiscale topographical patterns, composed of titanium dioxide nanocones and bismuth oxide nanodots, enhance osteogenic differentiation of mesenchymal stem cells by inducing mechanotransduction, as evidenced by the presence of abundant long lamellar protrusions and filopodia. The heterojunction forms a built-in electric field at the nanoscale interface of Ti implants, which can promote osteogenic differentiation of mesenchymal stem cells via the PI3K signalling pathway mediated by intracellular calcium ions. The research provides a new resolution for enhancing bone regeneration via tuning the electro-mechanobiological microenvironment of the bone implant (Fig. 8). Subsequently, the design was further refined by in situ discrete distribution of nano-heterojunctions on titanium oxide nanofibres to mimic the heterogeneous electrical microenvironment. The material has been shown to directly regulate calcium ion channelling, promoting anti-inflammatory macrophage polarization and a pro-angiogenic phenotypic transformation, thereby enhancing neovascularization at the early stage of osteogenesis. Mechanistic studies indicate that FGF2 secretion mediated by the PI3K signalling pathway may partly explain the strengthened coupling of immunomodulation and angiogenesis and subsequent bone regeneration [285].

Beyond mechanical force–electric coupling, Fan et al. expanded the combined engineering of biophysical cues to include light–cellular force–electric coupling using piezoelectric heterojunctions [286]. An array of heterojunctions (TiO_2_/Bi_2_WO_6_) decorating TiO_2_ nanowires was fabricated as a biocompatible implant. Upon exposure to near-infrared light, the piezoelectric heterojunctions generated reactive oxygen species and heat, killing bacteria via photodynamic and photothermal therapy, respectively. On the other hand, the mechanical forces generated by stem cells grown on the nanotopographic implant triggered the heterojunctions to intrinsically produce electric fields, further promoting osteogenesis and facilitating osteointegration. The multifunctionality of nano-heterojunctions highlights their potential for pathological bone repair, for example under conditions such as diabetes and radiation-induced bone damage, which are prone to infection and impaired angiogenesis. Further in-depth research using well-developed disease models is needed to support this potential.

### Interfacial parameter–mechanism–outcome framework

Interfacial cues in bone regeneration can be more systematically understood through a parameter–mechanism–outcome framework. The studies investigating the synergistic effects of different surface topographies and electrical stimulation on bone regeneration are listed in Tables 2 and 3, according to this framework. Specifically, micro/nanotopographical features and electroactive interfaces act as key input parameters that regulate cellular behaviour via distinct yet partially convergent pathways. Topographical parameters, such as feature size, spacing, and roughness, primarily influence focal adhesion formation, cytoskeletal organization, and nuclear deformation, thereby activating mechanotransduction pathways. These processes are closely associated with integrin signalling, actomyosin contractility, and chromatin remodelling, ultimately guiding osteogenic differentiation.

In parallel, electrical parameters—including electric field strength, waveform, frequency, and stimulation duration—modulate membrane polarization and activate voltage-gated ion channels, particularly calcium channels, triggering downstream signalling cascades that regulate cell migration, proliferation, and differentiation. Importantly, these mechanotransduction and electrosensing pathways may converge at shared molecular nodes, such as ion-channel regulation and cytoskeletal remodelling, leading to coordinated biological outcomes, including osteogenesis, angiogenesis, and immunomodulation.

However, interpretation of these effects is often complicated by confounding factors, including variations in surface chemistry, substrate stiffness, wettability, and ion release from materials. In electroactive systems, additional factors such as Faradaic reactions, local pH changes, and reactive oxygen species (ROS) generation may also contribute to the observed biological responses. Therefore, careful decoupling of these parameters is essential for establishing a clear mechanistic understanding.

### Practical implementation and safety considerations of self-powered systems integrated with mechanical force

A critical prerequisite for the clinical translation of self-powered electrical stimulation systems for bone regeneration is the ability to sustain stable, physiologically relevant electrical output throughout the entire 4–12 week bone healing cascade, a parameter often overlooked in preliminary laboratory studies. To date, the stability of the electrical output has mostly been assessed after a period of in vitro degradation or during specific working cycles. For bioresorbable self-powered scaffolds, hydrophobic PLGA encapsulation combined with an island-bridge electrode structure effectively resists in vivo body fluid erosion, enabling triboelectric nanogenerator devices to maintain a consistent ~ 4 V voltage output for 8 weeks in rat fracture models, fully covering the critical early bone repair period without signal attenuation [287]. Additionally, a shape-memory piezoelectric nanogenerator (sm-PENG) maintains a steady 20 µA rectified short-circuit current even after 10,000 working cycles, resisting mechanical fatigue from bodily movement [288]. Moreover, an optimized 0.4% ZnO/RSF piezoelectric hydrogel stably generates a voltage of ~ 80 mV under physiological pressure, with no significant output loss after 4 weeks of in vitro degradation. The hydrogel also shows excellent fatigue resistance, retaining stable voltage output after 100 compression cycles under repeated mechanical stress [289].

While topography-mediated biophysical cues and exogenous electrical stimulation exhibit robust pro-osteogenic potential for bone repair and regeneration, the risk of over-stimulation and subsequent adverse cellular responses must be rigorously addressed in osteogenic niche design, as demonstrated by mechanistic studies of electrical and mechanical stimulation in neural and myogenic models. Excessive electrical stimulation, where due to excessively high field strength or prolonged exposure, disrupts cellular ion homeostasis and membrane dynamics. Dorsal root ganglion cells showed reduced viability, elevated apoptosis and senescence at 200 mV/mm (compared with the optimal 100 mV/mm for 1 h), while extended 2 h stimulation, even at the optimal intensity, reversed the pro-proliferative effects [290]. C2C12 myoblasts also displayed dose-dependent viability loss at electrical field strengths exceeding 1 V/cm, with 3.33 V/cm inducing significant cytotoxicity [2]. Mechanical over-stimulation elicits similar harm: 13% cyclic tensile strain (compared with the optimal 9%) in C2C12 cells caused marked cell death via cytoskeletal damage and failed strain-avoidance responses [291]. For bone repair, these preliminary findings can be translatable: osteoblasts and bone mesenchymal stem cells (BMSCs) rely on tightly regulated calcium signalling and cytoskeletal dynamics for osteogenic differentiation, and high-intensity crack-generated flexoelectricity or supra-physiological electrical/topographical cues would perturb these pathways. This remains speculative. The intensity range of electrical stimulation detrimental to osteogenesis requires further experimental validation and exploration.

**Table 2:** Overview of surface topography and electrically stimulating for non self-powered materials for bone defect repair

Materials	Topography metrics	Elecrical metrics		Cell type	Model type	Endpoint (in vitro)	Endpoints (in vivo)	Evidence level	Ref.
	Feature Size/roughness	Power source	Dose metrics for non-self-powered system						
Anodized nanotubular titanium	Nanotubes: diameter 40–60 nm; depth 200–250	Multi channel electrical stimulator	voltages = 1, 5, 10 and 15 V Waveform=Constant bipolar pulses Frequency = 20 Hz Duty/Pulse Width = 0.4 ms pulse Duration = 1 h/day	Osteoblasts	NA	Cell density, cell proliferation	NA	in *vitro*	[267]
Polyaniline/Col1/sulfated hyaluronan	nanofiber with mesoporous: pore sizes 200–300 nm	Transformer-like coupling system	voltages = 3.6mV/cm Waveform=rectangular pulses Frequency = 10 Hz Duty/Pulse Width=7ms/pulse Duration = 4 h stimulation/4 h pause	hMSCs	NA	ALP Activity, Calcium Concentrations, Osteogenic Markers expression	NA	in *vitro*	[66]
Type 1 collagen coatings with BGN	nanoparticles: diameter 300–400 nm	Transformer-like coupling systen	Electric field = 3.6mV/cm Waveform=rectangular pulses Frequency = 10 Hz Duty/Pulse Width=7ms/pulse Duration = 4 h stimulation/4 h pause	hMSCs	NA	ALP activity, Calcium deposition, Osteogenic gene expression	NA	in *vitro*	[268]
TiO2 nanotube/polyaniline	Nanotubes: pore diameter 51.96 nm	Endogenous bioelectricity	Charge Transfer Resistance = 173.26 Ω	MC3T3-E1	NA	Osteogenic differentiation markers, biominalization, antibacterial activity	NA	in *vitro*	[272]
PD@BP	Nanofibers: Fiber diameter ~ 2 μm	Endogenous bioelectricity	Electrical conductivity ~ 0.4 S/m	Schwann cells, BMSCs	Mouse critical-size skull defect model	Axon regeneration, neuropeptide and neurotrophic factor secretion, osteogenic differentiation	Bone volume fraction, neurogenesis, osteogenesis marker expression	in vivo	[273]

**Table 3:** Overview of surface topography and electrically stimulating for self-powered materials for bone defect repair

Materials	Topography metrics	Elecrical metrics		Cell type	Model type	Endpoint (in vitro)	Endpoints (in vivo)	Evidence level	Ref.
	Feature Size/roughness	Power source	Output metrics for self-powered systems						
Polarized BaTiO3 ferroelectric nanorod array on titanium	Nanorods: length 500–600 nm	Ultrasound- driven piezoelectricity (dynamic) and voltage polarization (static)	Surface potential −237 to 139 mV (dynamic); 530.8 mV(static). Piezoelectric coefficient = 136.93 pm/V	mBMSCsosteosarcoma cells	Mouse subcutane-ous tumor model, Rat femur defect model	Colony formation, spindle structure, ALP activity, mineralization, osteogenic gene expression	Tumor volume, survival rate, tumor apoptosis, bone volume fraction, bone mineral density, bone-implant contact	in vivo	[280]
Polarized BaTiO3 ferroelectric nanorod array on titanium	Nanorods: length 700–88 nm; cross section size 50 nm	Voltage polarization	Surface potential ~ 355mV; d33 = 249.1 pm/V	Dorsal root ganglion neurons; rBMSCs	NA	CGRP expression, ALP activity, osteogenic gene expression, calcium transients, transcriptomic profiling	NA	in *vitro*	[282]
Porous Ti6Al4V scaffold coated with BaTiO3 nanoparticles	Nanoparticles: Macropore Size 659 ± 52 μm; Nanoparticle diameter ~ 200 nm	Voltage polarization	Surface potential = 30.97 ± 2.32 mV; d33 = 0.7 pm/V	MSCs; HUVECs	Sheep cervical interbody fusion model	ALP activity, mineralization, osteogenic gene expression, calcium concentration, VEGF and PDGF-BB secretion,	Bone fusion, bone volume fraction, bone-implant contact, elemental Ca/P ratio, vascular volume, vascular surface area, vessel nength/diameter	in vivo	[283]
TiO2 nanocone with Bi2O3 nanodot heterojunctions on titanium	Nanodots: diameter ~ 5.0 nm	heterojunctions	Surface potential~110mV; Bi2O3 band gap ~ 2.1–2.8 eV; TiO2 band gap ~ 2.1–2.8 eV	BMSCs	Rat distal femur defect model	Mineralization, YAP localization, intracellular calcium, osteogenic gene expression	Bone volume fraction, trabecular thickness, bone area ratio	in vivo	[270]
PVDF with cobalt ferrite oxide nano particles	Nanoparticles: size 35–55 nm	Piezoelectric stimulation by a magnetic bioreactor	16 h stimulation period; 8 h nonactive period; d33 = 151 pm/V; alternating magnetic field (0 − 230 Oe); magnetization saturation = 18 kOe of 11.1 emu/g;	hMSCs	NA	Cytoskeleton dynamics, Metabolomic analysis (acids, lipids, nucleotides, and carbohydrates)	NA	in *vitro*	[277]
BaTiO3/P(VDF-TrFE) and hydroxyapatite(HAp)/P(VDF-TrFE) scaffolds	Nanoparticles: HAp nanoparticles < 200 nm; BTO nanoparticle size < 100 nm	Voltage polarization by corona poling method	Surface potential: HApP(VDF-TrFE) ~390mV; BTO/P(VDF-TrFE) ~270mV; d33: HApP(VDF-TrFE) ~13pm/V; BTO/P(VDF-TrFE) ~14pm/V	osteoblasts	mouse calvarial defect model	osteogenic mineralization, cell morphology, focal adhesion, and growth factor secretion	Bone volume/area (micro-CT) and histological evaluation (H&E, Masson’s trichrome)	in vivo	[278]
Osteoblast footprints based on PDMS with BaTiO3	Osteoblast footprints: cellular scale	Voltage polarization and dynamic mechanical stimulation by an equiaxial tensile machine	Surface potential after polarization=10mV; Surface potential after the bending strain=300mV	ADSCs	NA	Cell morphology and attachment, mineralization, ALP activity, osteogenic gene expression, and osteogenic protein expression	NA	in *vitro*	[279]
Piezoelectric BaTiO3 substrate	Micropillar: heights 20–80 μm; cross-sectional area 100 × 100 µm2; space of the pillar 100 μm	Polarization by corona poling and interaction with endogenous electronegative	d33 = 24.11 pm/V	rBMSCs	Rat femoral diaphysis defect model	Antibacterial efficiency, cell spreading, mechanosensing, osteogenic differentiation	Nascent bone formation, bone-implant contact, and bone volume/total volume	in vivo	[253]

## Limitations and future directions

While current research presents promising results, clinical translation of complex topographical and electroactive cues into practical, tissue-engineered bone grafts presents significant challenges. Specifically, there is often a lack of standardized protocols for acellularization and cell seeding, which is critical for the reliability of allogeneic and xenogeneic grafts. Also, the diversity of evaluation criteria used across studies makes it difficult to compare outcomes and establish clear regulatory approval pathways. In addition, the research periods of in vivo experiments were regularly limited to 1, 4 and 8 weeks. Thus, long-term stability and biocompatibility need to be monitored in future studies using preclinical large-animal experiments.

To fully replicate the structural complexity of native bone tissue, future research should move beyond static, simplified designs and instead focus on developing hierarchically structured biointerfaces integrated with dynamically tuneable electrical stimulation cues. Moreover, intelligent optimization strategies—such as gradient surface engineering integrated with statistical learning approaches—are urgently required to overcome the current limitations in property–function matching, thereby driving the progressive development of biointerface engineering for bone regeneration.

## Conclusion

Bone regeneration is a highly orchestrated, dynamic process involving osteoblasts, osteoclasts, osteocytes, and mesenchymal stem cells (MSCs), tightly regulated through intercellular signalling and niche-specific biophysical cues. Among these, interfacial interactions—particularly those arising from micro/nanoscale surface topography and local electric fields—play pivotal roles in directing cellular fate and promoting tissue remodelling. Recent advances in biomaterials design have emphasized the importance of biomimetic replication of native bone extracellular matrix (ECM) properties. Micro- and nanoscale topographical features influence stem cell elongation, nuclear morphology, chromatin architecture, and focal adhesion dynamics, thereby modulating mechanosensitive pathways crucial for osteogenic differentiation. Concurrently, the recreation of electroactive interfaces simulating endogenous bioelectric signals has been shown to alter transmembrane potentials, activate voltage-gated ion channels, and initiate calcium-mediated signalling cascades that further promote osteogenesis and angiogenesis.

Importantly, accumulating evidence suggests that combining topographical and electrical cues may provide complementary and potentially synergistic effects for bone regeneration, although current support is primarily derived from preclinical studies. These emerging strategies highlight the critical role of interfacial phenomena in orchestrating complex cellular behaviours during bone regeneration. From a colloid and interface science perspective, the development of multifunctional biointerfaces that integrate hierarchical topographies with dynamic electroactivity offers significant opportunities to uncover new fundamental insights into cell–material interactions and to advance next-generation therapeutic platforms for regenerative medicine.

## Data Availability

No datasets were generated or analysed during the current study.
